# Nuclear imaging of liposomal drug delivery systems: A critical review of radiolabelling methods and applications in nanomedicine

**DOI:** 10.1016/j.addr.2019.05.012

**Published:** 2019-03-15

**Authors:** Francis Man, Peter J. Gawne, Rafael T.M. de Rosales

**Affiliations:** a School of Biomedical Engineering & Imaging Sciences, King’s College London, St Thomas’ Hospital, London SE1 7EH, United Kingdom; b London Centre for Nanotechnology, King’s College London, Strand Campus, London WC2R 2LS, United Kingdom

**Keywords:** Nanomedicine, Drug delivery, Liposome, PET, SPECT, Nuclear imaging, Theranostics, [^18^F]FDP, 3-[^18^F]fluoro-1,2-dipalmitoylglycerol, [^18^F]SteP2, 1-[^18^F]fluoro-3,6-dioxatetracosane, %ID/g, percentage of the injected dose per gram of tissue, 2HQ, 2-hydroxyquinoline, 4-DEAP-ATSC, 4,4′-bis(3-(*N*,*N*-diethylamino)propyl)thiosemicarbazone, ABC, accelerated blood clearance, ADA, amino diatrizoic acid, BAT, 6-[*p*-(bromoacetamido)benzyl]-1,4,8,11-tetraazacyclotetradecane-*N*,*N*′,*N*′′,*N*′′′-tetraacetic acid, BMEDA, *N,N*-bis(2-mercaptoethyl)-*N’,N’*-diethyl-ethylenediamine, CB-TE2A, 4,11-bis(carboxymethyl)-1,4,8,11-tetraazabicyclo-(6.6.2)hexadecane, CuAAC, copper-catalysed azide−alkyne cycloaddition reaction, DFO, desferrioxamine, DISIDA, diisopropyl iminodiacetic acid, DSPE, distearoylphosphatidylethanolamine, DOTA, 1,4,7,10-tetraazacyclododecane-1,4,7,10-tetraacetic acid, DPPE, 1,2-dipalmitoyl-sn-glycero-3-phosphoethanolamine, DTPA, diethylenetriaminepentaacetic acid, HMPAO, hexamethylpropyleneamine oxime, HSA, human serum albumin, HYNIC, hydrazinonicotinic acid, IAL, ionophore-assisted loading, IgG, immunoglobulin G, IVIVC, *in vitro-in vivo* correlation, LAI, liposomal amikacin for inhalation, LDL, low-density lipoprotein, LE, labelling efficiencies, LOX-1, lectin-like oxidized low-density lipoprotein receptor-1, NODAGA, 1,4,7-triazacyclononane,1-glutaric acid-4,7-acetic acid, NTA, nitrilotriacetic acid, PEG, polyethylene glycol, PFS, patient progression-free survival, PLA, PEGylated liposomal alendronate, RCY, radiochemical yield, TCEP, tris(2-carboxylethyl)phosphine, TETA, 1,4,8,11-tetraazacyclotetradecane-1,4,8,11-tetraacetic acid, TSC, ^99m^Tc-sulfur colloid

## Abstract

The integration of nuclear imaging with nanomedicine is a powerful tool for efficient development and clinical translation of liposomal drug delivery systems. Furthermore, it may allow highly efficient imaging-guided personalised treatments. In this article, we critically review methods available for radiolabelling liposomes. We discuss the influence that the radiolabelling methods can have on their biodistribution and highlight the often-overlooked possibility of misinterpretation of results due to decomposition *in vivo*. We stress the need for knowing the biodistribution/pharmacokinetics of both the radiolabelled liposomal components and free radionuclides in order to confidently evaluate the images, as they often share excretion pathways with intact liposomes (*e.g.* phospholipids, metallic radionuclides) and even show significant tumour uptake by themselves (*e.g.* some radionuclides). Finally, we describe preclinical and clinical studies using radiolabelled liposomes and discuss their impact in supporting liposomal drug development and clinical translation in several diseases, including personalised nanomedicine approaches.

## Graphical abstract

**Figure:**
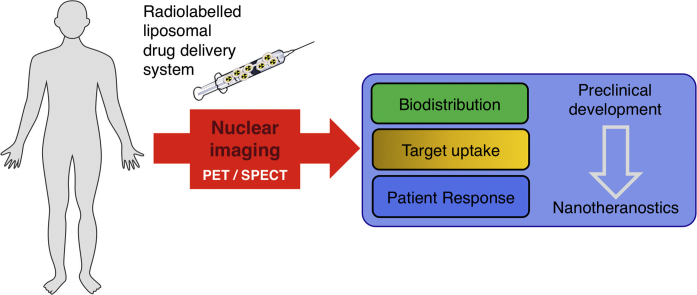

## 1 Introduction

Nanomedicine-based drug delivery aims to improve disease treatment by increasing the targeted accumulation of small-molecule drugs into diseased tissue while minimising systemic toxicity. Of the various drug delivery systems available, liposomes have had the most significant impact in clinical medicine to date, particularly in the field of anticancer drug delivery, with several products clinically available [1,2]. Many new liposomal drugs for other diseases (*e.g.* autoimmune, cardiovascular) are currently in clinical trials [2], and new exciting applications are emerging involving their combination with immunotherapies and radiotherapies [3,4].

In order to develop the best liposomal therapies possible, it is important to understand their *in vivo* behaviour. To achieve this, it is essential to develop non-invasive imaging techniques that allow us to visualise, quantify, and monitor their biodistribution over time and, ideally, provide information regarding drug release. Besides its clear role in the development of liposomal therapies, another factor where imaging drug delivery systems could play an important role in the future is the individualised prediction of therapeutic efficacy. This is particularly critical when we consider that the most common mechanism by which liposomal nanomedicines accumulate at target tissues (*i.e.* the enhanced permeation and retention effect or EPR), is a phenomenon that is highly heterogeneous in humans [5,6]. This heterogeneity has been blamed as one of the main factors responsible for the perceived low efficacy of nanomedicines in humans, compared to preclinical studies [7]. Thus, non-invasive imaging techniques that identify which patients or lesions will accumulate high concentrations of the nanomedicine at the intended target(s) could allow for highly efficacious personalised nanomedicinal treatments [8,9].

There are several imaging techniques available to image liposomal nanomedicines *in vivo*, each one having advantages and disadvantages for this purpose. For example, nanomedicines labelled with paramagnetic ions, such as Gd^3+^ or Mn^2+^, are detectable by magnetic resonance imaging (MRI) [10]. However, the low sensitivity of MRI (sensitivity defined here as the amount of label required to be detected by the imaging technique being discussed), low signal-to-background ratios achievable, and the dependence of the imaging signal on its microenvironment, makes whole-body detection and quantification complicated. Ultrasound imaging (US), despite its excellent spatial and temporal resolution, suffers from other disadvantages; particularly not allowing whole-body imaging and limited tissue imaging depth [11]. Computed tomography (CT) has been used to image liposomal nanomedicines at the whole-body level [12], but similarly to MRI, it suffers from low sensitivity and leads to high radiation doses, particularly when imaging the whole body. Labelling liposomal nanomedicines with optical labels such as fluorophores, allows imaging using techniques such as fluorescence molecular tomography (FMT) [13]. This technique allows high sensitivity and quantifiable *in vivo* biodistribution studies in animal models, but with limited applications in the clinical setting due to its low tissue penetration.

Nuclear imaging includes positron emission tomography (PET) and gamma-emitting techniques such as single-photon emission tomography (SPECT) and planar scintigraphy. These radionuclide-based techniques have near-ideal properties to image liposomal nanomedicines *in vivo*, in both animals and humans. In comparison with the previously discussed imaging methods it benefits from high sensitivity, whole-body capabilities, absence of tissue penetration issues, and accurate quantification. It is particularly important to highlight the high sensitivity of nuclear techniques in the context of imaging therapeutic nanomedicines. Thus, unlike modalities commonly regarded as insensitive such as MRI and CT that require the injection of gram quantities of contrast agents, nuclear imaging is achieved in humans with amounts of micrograms or less. As a consequence, imaging with a sub-therapeutic microdose of a liposomal nanomedicine is possible. This is a significant advantage over other imaging modalities in the context of facilitating their preclinical drug development and their potential clinical use in a theranostic approach to predict therapeutic efficacy. One limitation of nuclear imaging modalities is that their spatial resolution is in the range of 1–10 mm, depending on the instrument and radionuclide used (see Section 2), and is therefore lower than optical or MR imaging. Although this does not allow the visualisation of individual nanocarriers or cells, it is sufficient to measure the uptake of nanocarriers in organs and even their distribution within organs, particularly at the human scale.

In order to detect liposomal nanomedicines with nuclear imaging, these have to be modified by incorporation of a suitable radionuclide into their structure. In this review we aim to identify and discuss the different radiochemical methods that have been used to date to image and track the biodistribution of liposomal nanomedicines *in vivo*, as well as their applications in both animal and human studies. We will first briefly describe the main characteristics of radionuclide/nuclear imaging that make these techniques highly suitable for imaging drug delivery systems *in vivo*. In the following section we discuss the different choices of methods for radiolabelling and radionuclides, with particular emphasis on the stability of the resulting radiolabelled nanomedicines, and the potential for misinterpretation of results due to *in vivo* release of the radiolabel. In the last section we will discuss how these radiolabelling methods and products have been used to date to answer specific questions regarding the *in vivo* biodistribution of different liposomal nanomedicine formulations, their pharmacokinetics, and therapeutic efficacy in different preclinical disease models, as well as clinical examples. Finally, we will draw some conclusions and outline future perspectives of this exciting area of radionuclide imaging and nanomedicine.

## 2 Radionuclide imaging

Before we review the different liposome radiolabelling methods it is important to be aware of the mechanisms by which nuclear imaging techniques are able to locate and quantify radionuclides. The imaging of radionuclides can be performed with two techniques: single-photon emission computed tomography (SPECT) or positron emission tomography (PET). By ‘tagging’ or ‘labelling’ compounds with radionuclides (radiolabelling), these two techniques can be used to non-invasively track small molecules, macromolecules and cells inside the body and understand biological processes in real time within living organisms. Due to the detection of high-energy photons emitted by radionuclides, PET and SPECT have no tissue depth penetration limits and are also highly sensitive (10^-10^–10^-12^ M) compared to other imaging modalities such as MRI (10^-3^–10^-5^ M). Critically, as briefly mentioned above, these properties combined mean that imaging can be performed in humans and other animals, using such small amounts of compounds that they do not disturb the biological process being observed.

**Fig. 1:**
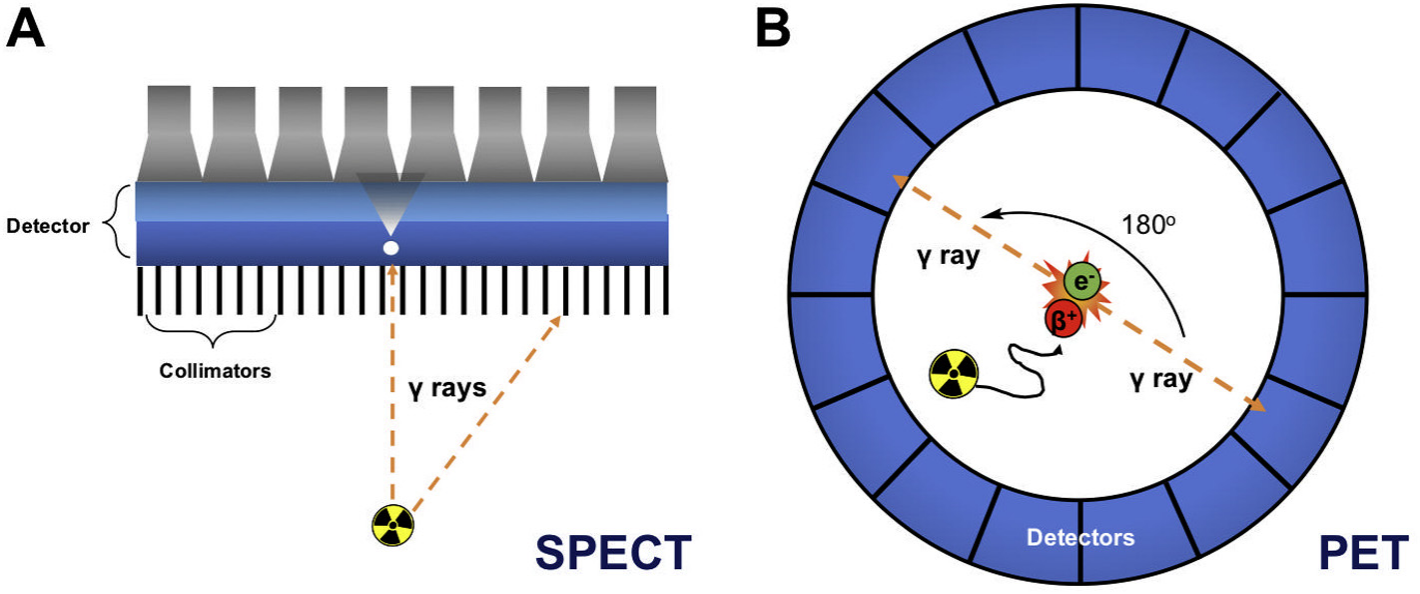
Schematic of the detection of radionuclides using **(A)** single-photon emission computed tomography (SPECT) and **(B)** positron emission tomography (PET).

Radionuclides that emit gamma ray photons at defined energy levels (Table 1) can be imaged using a gamma camera, creating a planar scintigraphic image. SPECT imaging is performed by rotating the camera around the subject to capture emissions in 3D. To determine the origin of the photons, collimators are used that exclude diagonally incident photons (Fig. 1A). PET, on the other hand, relies on radionuclides that decay by emitting positrons (Table 1, Fig. 1B). These interact with electrons in events known as annihilations that occur within a certain range of the radionuclide, depending on the positron energy (Table 1). This is known as the positron range, and for commonly-used radionuclides in PET it can be as low as 0.6 mm for ^18^F to as high as 2.9 mm for ^68^Ga, for example [14]. Each annihilation releases energy in the form of two 511 keV photons, emitted at an angle of approximately 180° from each other. PET cameras consist of a ring of detectors designed to detect these annihilation photons and pinpoint the precise origin of the annihilation event along the so-called ‘line of response’ and therefore the approximate location of the PET radionuclide (Fig. 1B). Because of this uncertainty about the position of the source of the positron, there is a fundamental limit to the spatial resolution achievable by PET. Consequently, better images can be obtained from PET radionuclides with low positron energy. Furthermore, because PET cameras rely on coincidence detection and do not require collimators, the sensitivity of PET is superior to that of SPECT [11]. In terms of spatial resolution, that of clinical SPECT scanners (5–12 mm) is slightly lower than with PET scanners (3–6 mm), however there is little difference in resolution between preclinical instruments (*ca.* 1 mm) [15]. PET also provides quantitative images. Despite this, imaging in the clinic using SPECT is often less costly and is performed more often than PET, most likely because of wider availability of SPECT isotopes and radiotracers, particularly those based on ^99m^Tc (*vide infra*), and SPECT scanners. There are, however, an increasing number of PET scanners and radiotracers becoming available in clinics worldwide, driven by their high sensitivity and spatial resolution compared to SPECT cameras. A particular advantage of SPECT over PET is the possibility of imaging multiple isotopes and therefore multiple radioactive compounds within the same subject. This is due to SPECT radionuclides having unique energy emissions that can be detected simultaneously and independently. In PET, however, all photons emitted during positron annihilation have the same 511 keV energy, making multi-radionuclide imaging not currently possible with standard scanners. Interestingly, many PET radionuclides also emit characteristic gamma rays, and it is therefore possible to simultaneously detect multiple PET isotopes with additional gamma-ray detectors by locating triple-coincidence events [16].

**Table 1:** Summary of the emission properties, half-lives and common applications of all radionuclides discussed in this review.

Radionuclide	Decay mode	Half-life	Imaging type	Common applications
Zr-89	β+ (23%, 0.9 MeV)	78.4 h	PET	Antibody, cell and nanomedicine labelling
Cu-64	β+ (39%, 0.19 MeV)	12.7 h	PET	Antibody, nanomedicine and peptide labelling, hypoxia radiotracers
Mn-52	β+ (29.4%, 0.24 MeV)	5.6 d	PET	Antibody and cell labelling
Ga-68	β+ (89%, 1.899 MeV)	68 min	PET	Peptide and small molecule labelling
Ga-67	EC	3.3 d	SPECT	Radionuclide therapy
Tc-99m	IT	6 h	SPECT	Peptide, small molecule and cell labelling, perfusion imaging
In-111	EC	2.8 d	SPECT	Antibody and cell labelling
Re-186	β- (92%)	3.7 d	SPECT	Radionuclide therapy
Re-188	β- (100%)	17 h	SPECT	Radionuclide therapy
I-123	EC	13.2 h	SPECT	Antibody labelling, thyroid imaging
I-124	β+ (25.6%)	4.2 d	PET	Antibody labelling, thyroid imaging
I-125	EC	59.4 d	SPECT	Antibody labelling, thyroid imaging
I-131	β- (100%)	8 d	-	Radionuclide therapy
F-18	β+ (96%, 0.25 MeV)	109 min	PET	Small molecule and peptide labelling, bone imaging
Bi-213	β- (97%)	45.6 min	-	Radionuclide therapy
Ac-225	α	9.9 d	-	Radionuclide therapy
Y-90	β- (100%)	64 h	-	Radionuclide therapy
Lu-177	β- (100%)	6.6 d	-	Radionuclide therapy
Gd-159	β-	18.5 h	-	Therapy

EC = electron capture; IT = isomeric transition.

**Fig. 2:**
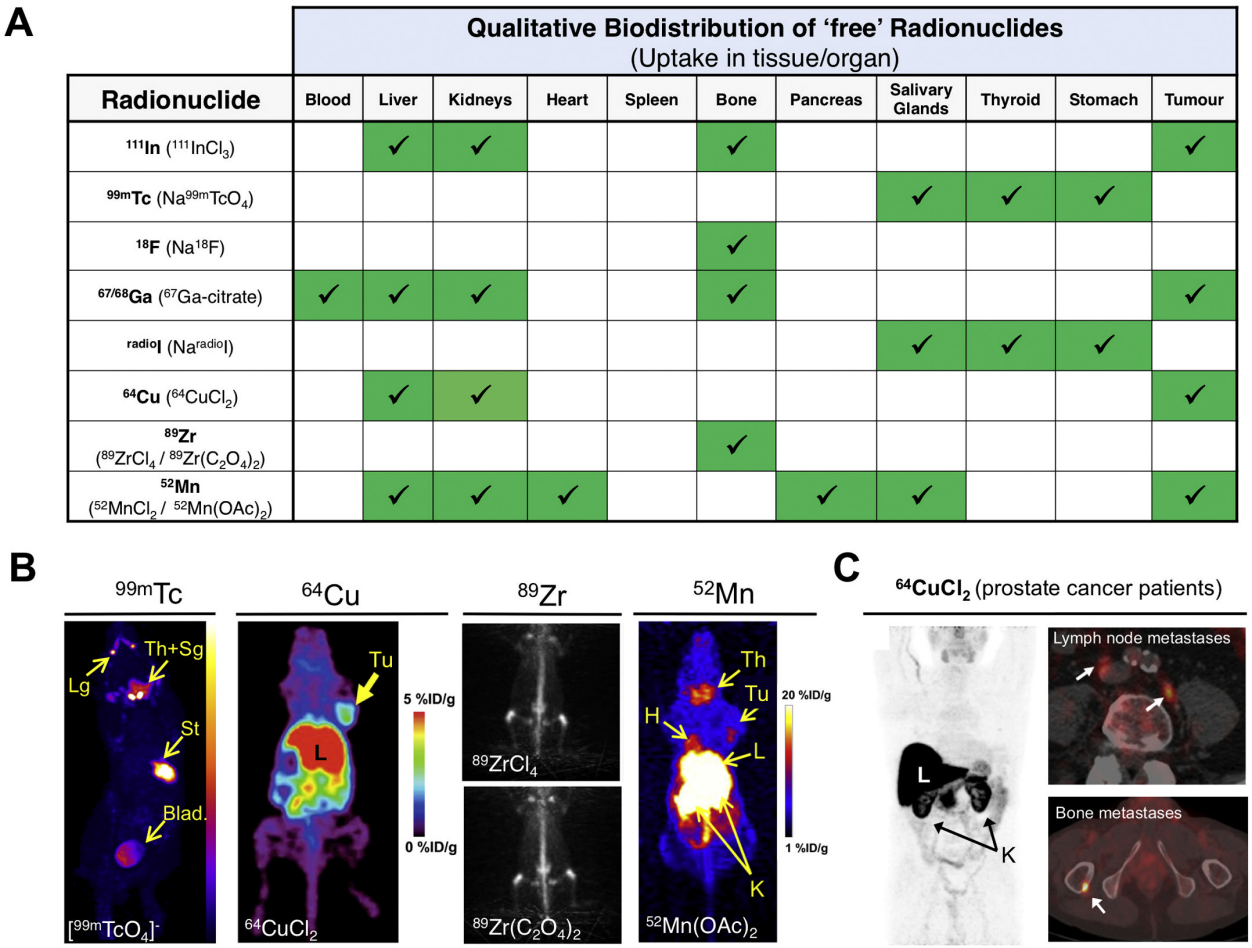
Biodistribution of ‘free’/unchelated radionuclides. **(A)** Uptake of ‘free’ radionuclides in various tissue/organs, including tumours. The actual chemical form administered is denoted in brackets (including ^111^In [22], ^99m^Tc [23], ^18^F [24], ^radio^Ga [25,26], ^radio^I [27], ^64^Cu [28,29], ^89^Zr [30], ^52^Mn [31]); **(B)** Representative mouse SPECT or PET images (maximum intensity projections) showing the biodistribution of ‘free’ ^99m^Tc, ^64^Cu (reprinted with permission from Peng et al. [32], Copyright 2006 SNNMI), ^89^Zr (reprinted with permission from Abou et al. [30], Copyright 2011 Elsevier) and ^52^Mn (reprinted with permission from Graves et al. [31], Copyright 2015 ACS). **(C)** PET maximum intensity projection image of ^64^CuCl_2_ biodistribution in a prostate cancer patient (reprinted with permission from Piccardo et al. [29], Copyright 2018 SNNMI). High uptake in the liver and kidneys can be clearly seen, as well as in prostate cancer metastases in lymph nodes and bone (white arrows). Lg: lacrimal glands; Th+Sg: thyroid and salivary glands; St: stomach; Blad: bladder; Tu: tumour; H: heart; L: liver; K: kidney.

The selection of a radionuclide for imaging purposes depends on various factors. First, it is important to understand the advantages and disadvantages of both nuclear imaging techniques as discussed above and choose one that will allow to obtain the maximum information from the envisaged studies. In clinical situations, if high spatial resolution and accurate quantification are important, PET should be the technique of choice. In preclinical situations, however, newer SPECT scanners often outperform PET in terms of spatial resolution. Most importantly, one should be aware that the half-life of the isotope should be in the same range as the biological half-life of compound being tracked/imaged. The labelling method needs to result in a biologically stable radiopharmaceutical, with a similar activity to the parent molecule in order to provide truly representative images. This is easier to achieve if the radionuclide can be attached with the least possible modifications to the structure of the parent compound. For example, small molecular weight compounds are often radiolabelled with ‘organic’ radionuclides such as ^18^F, ^11^C or radioiodine [17,18] to give radiopharmaceuticals with similar or even identical chemical structures. Alternatively, molecules can be radiolabelled using radiometals which require a chelator, which is a specific type of metal-binding molecule that provides stable radiometal conjugates [19]. The stability of the radiometal-chelator complex is critical to obtain representative images and therefore the choice of the pair should be carefully considered [20,21]. An important and often overlooked aspect is the biodistribution of the ‘free’, or unchelated, radionuclide (Fig. 2). Once *in vivo*, release of the radionuclide from the radiolabelled compounds can occur from metabolic reactions (*e.g.* enzymatic dehalogenation, macrophage degradation) or due to instability of the radiocomplex and competition from endogenous metals and chelators. The subsequent uptake of released radionuclide in tissues/organs, which may be indistinguishable from that of the parent nanomedicine, may lead to the misinterpretation of data/images. Based on these considerations, the different radionuclides and various methods of radiolabelling liposomes will now be explored, defined into groups, compared, and contrasted.

## 3 Radiolabelling liposomes

**Fig. 3:**
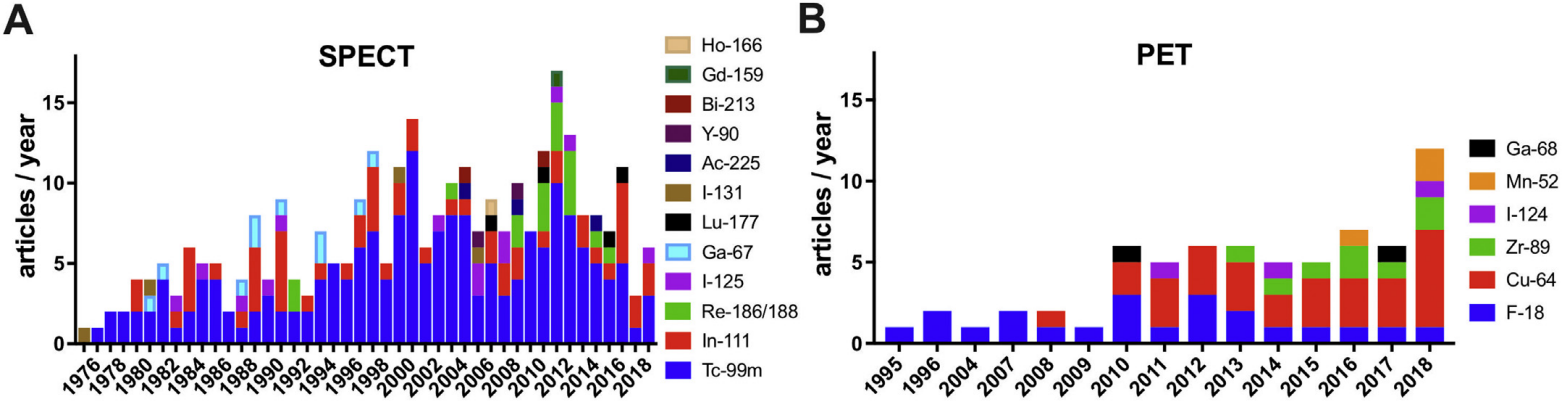
**(A)** Research articles published between 1973 and 2018 describing the use of gamma-emitting and therapeutic radionuclides for liposome labelling. For articles using more than one radionuclide, each radionuclide was counted as a separate publication. The total sum of publications in the graph (300) is therefore superior to the actual number of unique articles found (265); **(B)** Research articles published between 1995 and 2018 describing the use of positron-emitting radionuclides (PET) for liposome labelling. For articles using more than one radionuclide, each radionuclide was counted as a separate publication. The total sum of publications in the graph (67) is therefore superior to the actual number of unique articles found (59).

In this review we focus on liposomes [1], as methods and applications for radiolabelling other nanoparticle-based nanomedicines have been reviewed elsewhere [33,34]. Our review of the published literature in this area returned 322 articles with the earliest records from the early-1970s (see Supplementary Material for methodology). Technetium-99m (^99m^Tc) has been by a wide margin the most commonly used radionuclide to radiolabel liposomes (Fig. 3A), presumably because of its wide availability, low cost, favourable imaging properties, and a half-life (6 h, Table 1) that allows imaging for up to 24 h. ^111^In is the second most-used radionuclide, followed by radioisotopes of iodine. More recently, positron-emitting radionuclides such as ^18^F, ^52^Mn, ^89^Zr and particularly ^64^Cu have been increasingly used (Fig. 3B), reflecting the growing interest in PET imaging and the increasing availability of preclinical and clinical PET scanners.

**Fig. 4:**
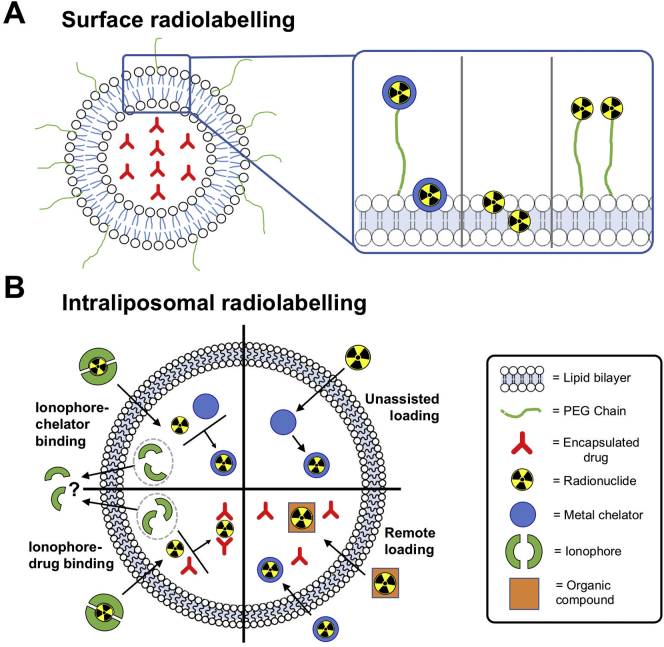
Schematic illustration of the different methods for radiolabelling liposomes. **(A)** Surface radiolabelling: the radionuclide, with or without a chelator, can be linked to the liposomal membrane via a PEG chain or incorporated directly into the lipid bilayer. **(B)** Intraliposomal radiolabelling: the radionuclide is encapsulated within the aqueous core. Ionophores can be used to transport radionuclides across the bilayer where they can be bound by chelators or drugs inside the liposomes, or radioactive compounds/complexes can passively cross the bilayer and become trapped.

After reviewing all these references, we classified the different liposome radiolabelling methods in the following two main categories (Fig. 4), based on whether the radionuclide is attached to components of the lipid bilayer (Fig. 4A), or the intraliposomal space (Fig. 4B).

### 3.1 Surface labelling

**Fig. 5:**
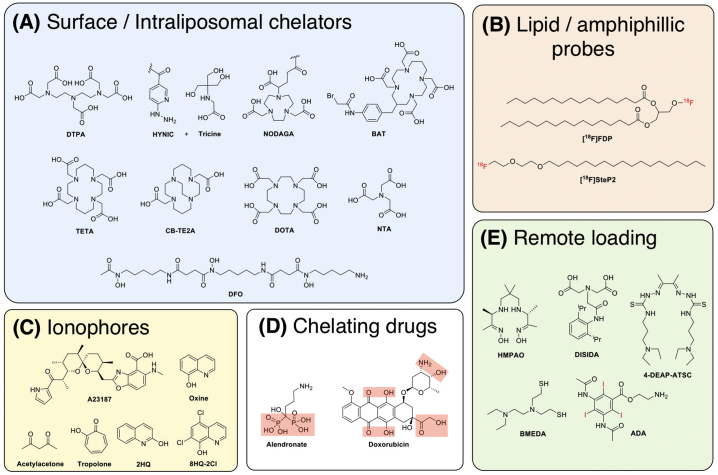
Schematic showing the chemical structures of various compounds used to assist the radiolabelling of liposomes, all of which are discussed in this review. **(A)** Structures of metal chelators that are either attached to the lipid surface or encapsulated inside the liposomal core, or **(B)** radiolabelled amphiphilic probes can be inserted into the lipid bilayer for radiolabelling. **(C)** Alternatively, ionophores can used to transport radionuclides inside the liposomal core and release the isotopes where they can either be trapped by binding to entrapped chelators or in some cases can bind directly to **(D)** the chelating groups of encapsulated drugs. **(E)** Radiolabelling can be also achieved by the remote loading of metal complexes or radio-iodinated compounds that become trapped in the liposomal core via protonation of the ligand used.

One of the most common methods to radiolabel liposomes is by inserting radionuclides into the lipid bilayer, otherwise known as surface labelling (Fig. 4A). The first example of this method was reported by Richardson et al. who showed that the surface of a liposome can be directly labelled with ^99m^Tc after reduction of ^99m^TcO_4_^-^ using stannous chloride (SnCl_2_) as a reducing agent [[35], [36], [37], [38], [39]]. To the best of our knowledge, there are no data on the exact binding site. One possibility is chelation by the phosphonate groups on the liposome phospholipid surface. Labelling efficiencies (LE) of >97% could be achieved after incubating for just 15 min at room temperature. However, labelling with this method was shown to be unstable *in vivo* [36,40]*.* Alternatively, surface labelling can be achieved by incorporating an appropriate chelator onto the liposome surface, either attached to the phospholipid or, in the case of long-circulating liposomes, to the PEGylated phospholipids (Fig. 4A). One of the earliest examples of this approach was reported by Hnatowich et al. who labelled liposomes with ^67^Ga and ^99m^Tc by chelation with diethylenetriaminepentaacetic acid (DTPA, Fig. 5A) conjugated to stearylamine, a long-chain hydrocarbon, allowing integration of this lipophilic molecule into the lipid-bilayer [41]. Similar subsequent work used liposomes pre-formulated with DTPA conjugated to the phospholipid on the liposome surface to bind to ^99m^Tc after reduction by stannous chloride. However, low serum and *in vivo* stability was observed using this method [[42], [43], [44]]. Later, Laverman et al. reported an improved method of radio-labelling PEGylated liposomes containing hydrazinonicotinic acid (HYNIC, Fig. 5A) conjugated to the lipid distearoylphosphatidylethanolamine (DSPE). HYNIC is a highly efficient Tc chelator that, in combination with co-ligands such as tricine (Fig. 5A), allows radiolabelling with high-specific activities [45]. The labelling efficiency was >95% after 15 min incubation at room temperature, which meant that no further purification was required – hence simplifying the labelling procedure. The ^99m^Tc-labelled liposomes showed high *in vitro* and *in vivo* stability [[46], [47], [48], [49]]. More recently, Varga et al. developed a new surface labelling method in which liposomes were formulated with 2-iminothiolane that could react with the widely used ^99m^Tc-tricarbonyl complex [50]. Whilst the labelling yields (90–95% LE) and stability are comparable to previous methods, the additional step required to convert ^99m^Tc-pertechnetate (^99m^TcO_4_^-^) to ^99m^Tc-tricarbonyl (^99m^Tc(CO)_3_^+^) could be seen as needlessly complex compared to other methods, particularly for human imaging studies. Several studies have since revisited using DTPA-conjugated liposomes radiolabelled with ^99m^Tc; DTPA was either conjugated to the phospholipid, DSPE [51], PEGylated DSPE [52], or to cholesterol during formulation of the liposomes [53].

Despite the high number of studies using ^99m^Tc for liposome tracking, it is worth nothing that its relatively short half-life (*t*_1/2_ = 6 h, Table 1) can limit its use in tracking liposomes to approximately 24 h post-administration. To overcome this limitation, particularly for long-circulating liposomes that exploit the EPR effect such as those used for cancer/inflammation therapy (*vide infra*), other studies have focused on surface labelling with DTPA using the longer-lived SPECT isotope ^111^In (*t*_1/2_ = 2.8 d). This was first described by Elbayoumi et al., however the required purification step by overnight dialysis is a serious limitation to their method [54], which could be overcome nowadays by using faster size-exclusion techniques such as those based on centrifugal filters. Other methods involved the use of non-radioactive indium metal in the radiolabelling procedure to saturate the DTPA chelators on the liposomal surface [[55], [56], [57]]. However, several reports have shown that LE of >95% can be achieved simply by incubating ^111^In with these formulations at 25–37 °C for up to 1 h [[58], [59], [60], [61]]. Interestingly, a direct comparison of the radiolabelling of DTPA-functionalised PEGylated liposomes with both ^99m^Tc (using both ^99m^TcO_4_^-^ and ^99m^Tc(CO)_3_^+^) and ^111^In was reported by Helbok et al. [58]. Labelling efficiencies of >95% were achieved with ^111^In over a wide liposome concentration range. Labelling over the same concentration range was possible with ^99m^TcO_4_^-^, however the LE was consistently lower (74.9 ± 6.2%), whereas for ^99m^Tc-carbonyl >80% LE was achievable but only with 50-fold more liposomes. Despite this, serum stabilities after 24 h for ^111^In and ^99m^Tc-carbonyl were comparable and their *ex-vivo* biodistribution in Lewis rats similar over 12 h. Uptake in the kidneys after 12 h was more than 2-fold higher for ^99m^Tc-carbonyl-DTPA liposomes compared to ^111^In-labelled liposomes [58], suggesting the potential release of the radionuclide in a hydrophilic form. The authors also demonstrated radiolabelling with ^68^Ga and the therapeutic isotope ^177^Lu using the same formulation; achieving >95% LE for ^68^Ga-DTPA and >80% LE for ^177^Lu-DTPA, albeit using a 5-fold higher concentration of NP [58]. Surface labelling using DTPA has also been reported for the therapeutic radionuclides yttrium-90 [62], and holmium-166 [63].

More recently, reports have started to focus on surface labelling using chelators for PET radiometals. Malinge et al. used 1,4,7-triazacyclononane,1-glutaric acid-4,7-acetic acid (NODAGA, Fig. 5A) attached to PEGylated lipids to label magnetic liposomes with ^68^Ga, which could be purified with a magnetic column, however it is unclear if the use of such a short-lived isotope was justified in this context [64]. Labelling using ^64^Cu has been an increasingly popular choice, because its half-life allows tracking liposomes for up to *ca.* 48 h. Seo et al. were the first to describe a reliable method for the attachment of ^64^Cu to the surface of liposomes [[65], [66], [67], [68], [69]]. They synthesised a PEGylated lipid containing the ^64^Cu-specific chelator, 6-[*p*-(bromoacetamido)benzyl]-1,4,8,11-tetraazacyclotetradecane-*N*,*N*′,*N*′′,*N*′′′-tetraacetic acid (BAT, Fig. 5A). When inserted into the liposomal surface, this platform allowed LE of >80% after incubation at room temperature for 1 h, with >90% of the radiation still bound after incubation with mouse serum for 48 h. *Ex vivo* biodistribution 48 h after administration showed high splenic uptake of the liposomes compared to ^64^CuCl_2_ and the ^64^Cu-PEG-lipid suggesting *in vivo* stability of the formulation. Interestingly, the authors also showed ^64^Cu-PEG-lipid uptake in the liver was roughly 3-fold higher than the liposomes [65]. This uptake of radiolabelled lipids should be carefully considered when tracking liposomes as release of these structures may occur after uptake in tissues and subsequent destruction of liposomes. Additional work by Seo and collaborators looked at labelling by attaching ^64^Cu complexes of 1,4,8,11-tetraazacyclotetradecane-1,4,8,11-tetraacetic acid (TETA, Fig. 5A) and 4,11-bis(carboxymethyl)-1,4,8,11-tetraazabicyclo-(6.6.2)hexadecane (CB-TE2A, Fig. 5A) conjugated with 2-pyridyldithiol groups to maleimide functionalised liposomes [70]. After complexation with the radionuclide the complexes were activated using tris(2-carboxylethyl)phosphine (TCEP) to give the free thiol group, which would in turn allow binding to the liposome surface. Optimised conditions allowed >90% LE with >84% stability in mouse serum after 48 h. However, quenching with ethanethiol was performed first *in lieu* of having thiol reactive groups covering the liposome surface, which would likely affect the biodistribution. Intriguingly, the authors showed that attaching the complex to either PEG or non-PEGylated lipids altered the biodistribution, with 5% higher hepato-splenic uptake occurring after 48 h [70]. This work shows that the biodistribution of radiolabelled liposomes can easily be altered solely based on the position of the radiocomplex, which could be viewed as a drawback to surface labelling of liposomes. This method was also used to show that simply using DPPE, *i.e.* shortening the carbon chain length of the maleimide lipid by two units, caused a severe reduction in stability, with blood clearance decreasing from 18 h to 5 h [71].

**Fig. 6:**
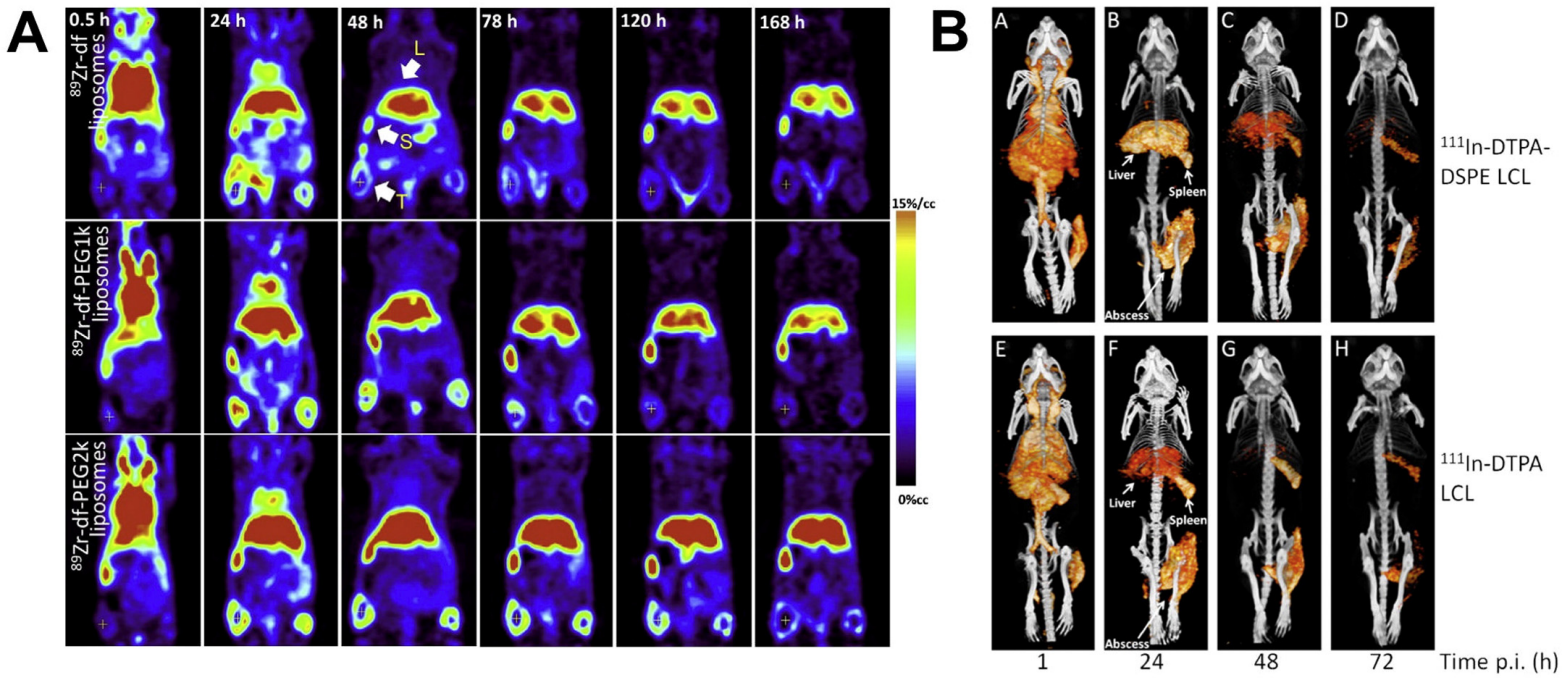
Small differences in the radiolabelling method can affect the biodistribution of liposomes. **(A)** Significantly higher EPR-mediated tumour and liver uptake and retention observed when using longer PEG chain lengths between the ^89^Zr chelator (DFO) and the liposomal surface (shortest on top, longest at the bottom). **(B)** Significantly higher liver uptake observed over time for liposomes labelled on the surface with ^111^In-DTPA (top row) compared to intraliposomally labelled liposomes using oxine and encapsulated DTPA (bottom row). EPR-mediated uptake in the infected tissue was not significantly different. Figures adapted with permission from (A) Seo et al. [81] and (B) Van der Geest et al. [60], Copyright 2015 Elsevier.

Work from other groups has focused on using DOTA (Fig. 5A) conjugated lipids for ^64^Cu labelling [[72], [73], [74], [75]]. Labelling efficiencies of 76–99% have been reported after incubation with mild heating (37–50 °C), with serum stability >95% after 24 h [73,74]. Jensen et al. compared surface-bound DOTA radiolabelled with ^64^Cu and the longer-lived isotope ^52^Mn [75]. ^52^Mn-DOTA liposomes were shown to have a shorter blood half-life, although this was not significant. Additionally, urinary bladder uptake was higher for ^52^Mn-DOTA liposomes for all timepoints after 40 min suggesting the ^52^Mn complex was less stable. Luo et al. described a method of radiolabelling porphyrin-phospholipid liposomes with ^64^Cu, in which the radionuclide was able to bind to the porphyrin chelator within the lipid bilayer [76]. Radiolabelling was shown to be dependent on the presence of the porphyrin, with low labelling efficiency (<20% LE after 4 h). Other groups have focused on surface labelling with ^89^Zr that has a half-life comparable to that of ^111^In, and is a better match for long-circulating PEGylated liposomes. It was previously shown by Abou et al. that chelator-free labelling with ^89^Zr was possible via binding of the radionuclide directly to the lipid phosphate head groups, however, this interaction was shown to be weak, contributing to low serum and *in vivo* stability [77]. To overcome this, several groups have performed surface labelling with ^89^Zr using desferrioxamine (DFO, Fig. 5A) as a chelator [[78], [79], [80], [81], [82]], which allows radiolabelling at neutral pH with only mild heating. Pérez-Medina and collaborators reported and compared two radiolabelling methods using this ligand; DFO was either attached directly to the surface and then radiolabelled, or the radio-complex synthesised and then attached to the liposomes using click-chemistry [78]. Using surface-bound DFO was shown to be superior to the latter method, with shorter radiolabelling times (4 h and 16 h, respectively), higher serum stability after 24 h (90% and 83%, respectively) and more favourable *in vivo* properties. The circulation time of the click-labelled liposomes was severely reduced with a blood half-life of 1.2 h, compared to 7.2 h for the surface-DFO liposomes, which the authors stated was due to higher tendency of the liposomes to aggregate. This resulted in higher clearance through the reticuloendothelial system (RES) and therefore lower overall tumour uptake of this formulation. Hence, only the surface-DFO labelling technique was used in later studies [79,80]. Seo et al. compared the effect of increasing PEG-length between the liposomal surface and the ^89^Zr-DFO complex [81]. They synthesised three formulations with DFO either bound directly to the lipid or with a 1K or 2K PEG spacer which showed no significant differences in terms of %LE, serum stability or blood half-life. However, image-based analysis showed significantly higher tumour uptake and retention over 168 h when using a 2K PEG spacer, as well as significantly higher liver and spleen uptake from 48 to 168 h compared to the other two formulations (Fig. 6A). This again highlights how small modifications in chelator position within the surface of radiolabelled liposomes can affect their biodistribution and pharmacokinetics.

The other major approach for surface labelling of liposomes involves non-metallic radionuclides covalently bound to both PEGylated/non-PEGylated lipids (Fig. 4A). Radiolabelling without the use of often bulky chelators can be beneficial, as this can affect the biodistribution of the liposomes as previously discussed. A small set of studies has looked at using radioisotopes of iodine with half-lives compatible with liposome tracking (Table 1). Kao et al. reported ^131^I-radiolabelled micelles [83], and two reports looked at using ^125^I for the tracking of liposomes conjugated with monoclonal antibodies. In both cases, the radioiodine was bound to the antibody attached to the surface, with the liposomes additionally radiolabelled using an internalised ^99m^Tc complex [84] or a surface-bound ^111^In-DTPA complex [61]. Other work has focused on surface labelling using the shorter-lived PET radionuclide ^18^F. Several groups have described ^18^F-based surface labelling using 3-[^18^F]fluoro-1,2-dipalmitoylglycerol ([^18^F]FDP, Fig. 5B) [[85], [86], [87], [88]]. The precursor containing a reactive tosyl leaving group could be reacted with K[^18^F]F/Kryptofix to give [^18^F]FDP within 20 min at 100°C, which was then mixed with a lipid formulation during liposomal preparation. Radiolabelled long-circulating liposomes could be prepared in just over an hour with a decay-corrected radiochemical yield of 70%. *In vivo* stability was shown with activity circulating in the blood and no observable bone uptake (a consequence of defluorination), at least within the timeframe of the imaging study (90 min) [85]. Similarly, Jensen et al. described a method using a radiolabelled cholesteryl ether [89]. After attachment of ^18^F, the compound was added during the formation of the liposomes resulting in >95% incorporation. An alternative method was reported by Urakami et al. using the amphiphilic probe, 1-[^18^F]fluoro-3,6-dioxatetracosane ([^18^F]SteP2, Fig. 5B) [[90], [91], [92], [93]]. Once synthesised, preformed liposomes could be radiolabelled using a solid phase transition method wherein the PET probe was transferred to a glass vial, and the solvent removed, the liposomes were added with agitation allowing the long alkyl chain to intercalate with the lipid bilayer on the liposome surface. This technique allowed both a labelling efficiency and stability in serum (after 30 min) of >80% [90] and the ability to label preformulated liposomes is very beneficial. Considering that liposomes and similar compounds in the nanometre scale tend to have long biological half-lives, it is easy to dismiss the use of ^18^F based on its short half-life, however it may be beneficial in applications where long-term tracking is not needed. This could include, for example, fast liposome trafficking to the brain [[91], [92], [93]] or accumulation in the heart [86,87] within an hour of administration. Another interesting example of this approach was reported by Rösch and collaborators, using ^18^F-radiolabelling to test the effect of linear and branched lipids on liposome distribution within the first hour after administration [94,95]. The lipids were radiolabelled via the copper-catalysed azide−alkyne cycloaddition reaction (CuAAC) between alkyne-functionalized lipids and a ^18^F-labelled azide compound and then added during synthesis of the liposomes. *In vivo* tracking of liposomes using this method was able to elucidate vast differences in biodistribution and accumulation within the first hour. Whilst maybe not applicable for longitudinal imaging of liposomes, labelling and tracking with ^18^F may be a valuable tool in the development of new formulations.

### 3.2 Intraliposomal labelling

As alternatives to radiolabelling the surface of a liposome, there are various methods to incorporate and trap radionuclides inside the liposomal core (Fig. 4B). This approach, in principle, should benefit from improved *in vivo* stability due to the protective effect of the lipid bilayer that prevents interaction between the radionuclide and the extra liposomal biological components (*e.g.* blood proteins, etc). In addition, the lack of surface modifications should result in identical physicochemical properties compared to the starting liposome. Some of the earliest studies performing the radiolabelling of liposomes achieved this by simply encapsulating a radiometal complex with DTPA inside the liposomal core during formation of the liposomes. This was first done with ^99m^Tc [[96], [97], [98], [99]], and later ^111^In [100] and ^159^Gd-DTPA [101] – as well as with the therapeutic isotope ^225^Ac by encapsulating the DOTA complex [102]. Alternatively, encapsulated drugs could themselves be labelled with radioiodine [56,[103], [104], [105], [106], [107], [108], [109]] or ^18^F [110] before liposomal formulation and more recently liposomes were radiolabelled by being prepared in the presence of [^18^F]FDG [[111], [112], [113], [114]]. Much like the surface-labelled liposomes that are radiolabelled during liposomal formulation, these techniques can be limited due to the longer, more complicated radiosynthesis needed (especially when using short-lived isotopes) as well as the inability to label preformed liposomes. However, the ability to directly label the drug inside the liposomes and track its distribution is clearly valuable and will be discussed further in Section 3.2.3 (*vide infra*). The following sections will focus on intraliposomal labelling techniques that allow the radiolabelling of pre-formed liposomes either with or without modification.

#### 3.2.1 Ionophore-chelator binding

The most common form of intraliposomal labelling is often achieved via the use of ionophores, which are molecules that allow the transport of metal ions (in this case radiometals) across lipid bilayers – often in the form of a neutral, lipophilic complex. Due to the metastable nature of these complexes, the radiometal can then be released inside the liposomes and bind to an entrapped chelator, forming a stable complex within the liposomal core (Fig. 4B). The first example of this was reported by Gamble and collaborators who embedded the calcium ionophore A23187 (Fig. 5C) into the lipid bilayer of liposomes, allowing transport of ^111^In inside the liposomal core where it was chelated by encapsulated nitrilotriacetic acid (NTA, Fig. 5A) allowing >90% LE [115,116]. Hwang et al. later reported several methods that did not require pre-formulating liposomes to incorporate an ionophore in the bilayer: ^111^In could be transported into NTA-containing liposomes by small molecular weight ionophores, 8-hydroxyquinoline (oxine, Fig. 5C) [117,118] as well as acetylacetone (Fig. 5C) [119] and tropolone (Fig. 5C) [120]. Additionally, Utkhede et al. later showed that DTPA-containing liposomes could be labelled by reacting ^90^Y with A23187, allowing transport of the complex across the bilayer [121]. Oxine was later used by Gabizon et al. to label liposomes encapsulating the chelator DFO with ^67^Ga [122,123], showing that when using tropolone as an ionophore the LE was threefold lower than with oxine. This technique using DFO was later adapted by Boerman et al. using ^111^In [124,125]. Similarly, Harrington and collaborators reported using ^111^In-oxine to radiolabel liposomes containing DTPA, which allowed >90% LE after 15 min incubation and high serum stability for up to 10 days [[126], [127], [128]]. The biodistribution of the radiolabelled liposomes with ^111^In-DTPA showed the long circulating properties of the PEGylated nanoparticles with high amounts of activity in the blood up to 24 h, followed by hepato-splenic uptake after that time – whereas ^111^In-DTPA was cleared rapidly [127]. Boerman et al. later showed that this labelling method was compatible with using an encapsulated drug. PEGylated liposomal prednisolone [129] and liposome encapsulated superoxide dismutase [100,130] could still be radiolabelled with >85% LE, albeit with a longer incubation time with ^111^In-oxine than previously reported. This method was later used to label liposomes with ^177^Lu by Wang et al. [131]. Van der Geest et al. later compared this labelling technique with surface labelling with ^111^In using DTPA-DSPE liposomes – the labelling of empty liposomes (without DTPA) was also reported [60]. Labelling efficiencies >95% were reported using both radiolabelling methods, as well >95% serum stability after 48 h, whereas the empty liposomes showed lower LE (62%) and serum stability (68 %). A DTPA challenge assay showed that the surface-labelled liposomes had a higher stability than oxine-DTPA and empty liposomes (93%, 46% and 2% respectively) after incubation with 10^-3^ M DTPA for 24 h. Interestingly, when assessing the *in vivo* distribution of the formulations in mice, the surface-labelled liposomes showed significantly higher liver uptake over 72 h – compared to the oxine-DTPA liposomes – whereas no difference was seen in spleen or the target abscess uptake (Fig. 6B) [60]. This may indicate that release of ^111^In-DTPA from inside the liposomes is occurring, suggesting lower *in vivo* stability, as ^111^In-DTPA is rapidly cleared [127], whereas ^111^In-DTPA-DPSE (released from liposomes during degradation) will likely accumulate in the liver.

The first use of this labelling strategy with a PET radionuclide was developed by Petersen et al. [132,133], and later used by Locke et al. [134], who used the ionophore 2-hydroxyquinoline (2HQ, Fig. 5C) to transport ^64^Cu across the liposomal bilayer where it can be trans-chelated with encapsulated DOTA. Labelling efficiencies >95% could be achieved after incubating the DOTA liposomes with the ionophore-complex for up to 1 h at temperatures between 20–50°C, with >99% serum stability after 24 h. The *in vivo* stability was shown via the long circulation time of ^64^Cu-liposomes compared to the free ^64^Cu-DOTA complex, which was cleared rapidly. The work also highlighted the intraliposomal pH as a key consideration when using this technique. Liposome loading in this instance was >95% and 70% for pH 4 and 5.9 respectively, suggesting the complexation by DOTA was affected [132]. This concept was explored further by Jensen et al. who used oxine derivatives to load ^52^Mn into DOTA encapsulated liposomes [75]. Labelling efficiencies above 90% could be achieved when using oxine and 5,7-dichloro-8-hydroxyquinoline (8HQ-2Cl, Fig. 5C) after incubation at 55°C with an intraliposomal pH 4, but increasing the pH to 7.8 led to a large reduction in labelling using oxine (ca. 30–70% LE) whereas this was not observed for 8HQ-2Cl. Therefore, the internal pH will not only affect the chelation by the internalised ligand, but also the dissociation of the ionophore complex used. The authors also compared liposomes labelled with ionophores to those labelled using surface-bound DOTA. The intraliposomally labelled ^52^Mn-liposomes showed a significantly higher blood half-life and lower urine activity was observed 5 h after administration, suggesting higher stability than the surface-labelled counterpart [75]. The use of oxine, as well as A23187, to radiolabel DOTA-containing liposomes was also reported by Sofou and collaborators with ^225^Ac [135,136]. Further work using ionophore-to-chelator labelling was reported by Li et al. who used oxine and 2HQ with ^89^Zr to label liposomes encapsulating DFO [137]. Labelling efficiencies of >95% and 83% were achieved using oxine and 2HQ respectively, with 94% stability in mouse serum after 48 h. Despite this, the *in vivo* instability of the ^89^Zr labelled liposomes was demonstrated by large bone uptake observed both 24 h and 48 h after administration, with little spleen or liver accumulation.

#### 3.2.2 Unassisted loading

The use of a chelator to transport a radiometal across the lipid bilayer of liposomes may not always be necessary. In the specific case of ^64^Cu^2+^, Henriksen et al. showed that simply incorporating a DOTA chelator inside of a variety of liposomal formulations was sufficient to achieve labelling efficiencies of over 90% in only 30 min [[138], [139], [140], [141]]. This ‘unassisted loading’ (Fig. 4B) of the radionuclide occurs due to depletion of intraliposomal non-radioactive copper by the DOTA chelator. A steep copper gradient is established across the membrane, causing diffusion of ^64^Cu^2+^ into the liposome where it is trapped upon chelation by the DOTA ligand. Not only does this technique increase the simplicity of radiolabelling liposomes, but it removes the need for ionophores, which are known to have a variety of biological activities [142]. Liposome labelling in this manner was found to be temperature-dependent with mild heating to 55°C needed to ensure efficient radiolabelling compared to the ionophore-assisted loading (IAL), which was temperature-independent. Therefore, the labelling of more temperature-sensitive liposomal formulations may be limited with this technique. Additionally, the need for pre-formulating liposomes with a chelator may again limit its use with liposomal nanomedicines already on the market. However, the usefulness of this technique for investigating new formulations and the *in vivo* distribution should not be downplayed.

#### 3.2.3 Ionophore-drug binding

Building on the previous work using radio-ionophore complexes, our group developed a facile method for the radiolabelling of liposomal formulations without the need for incorporated chelators and therefore without having to chemically modify the formulation [[143], [144], [145]]. This is based on the metal-chelating properties of certain drugs (Fig. 5D) that are able to bind the radionuclide after ionophore-mediated transport across the lipid bilayer (Fig. 4B). For example, complexes of doxorubicin with manganese have been previously reported [146,147]. High radiolabelling yields of Doxil® and pre-formed PEGylated liposomal alendronate (PLA) were easily achieved with the PET radionuclides ^89^Zr and ^52^Mn using oxine as an ionophore. The radiolabelling of the same formulations was also carried with ^64^Cu using 2HQ, however, quantitative labelling was only seen with PLA and only at higher drug concentrations. Since 2HQ has been established as a good ionophore for copper [132], this difference in radiolabelling is likely due to weaker drug-metal binding. Hence, the extent of radiolabelling using this method will always be limited by the interaction between the radiometal and the drug inside the liposomal formulation.

**Fig. 7:**
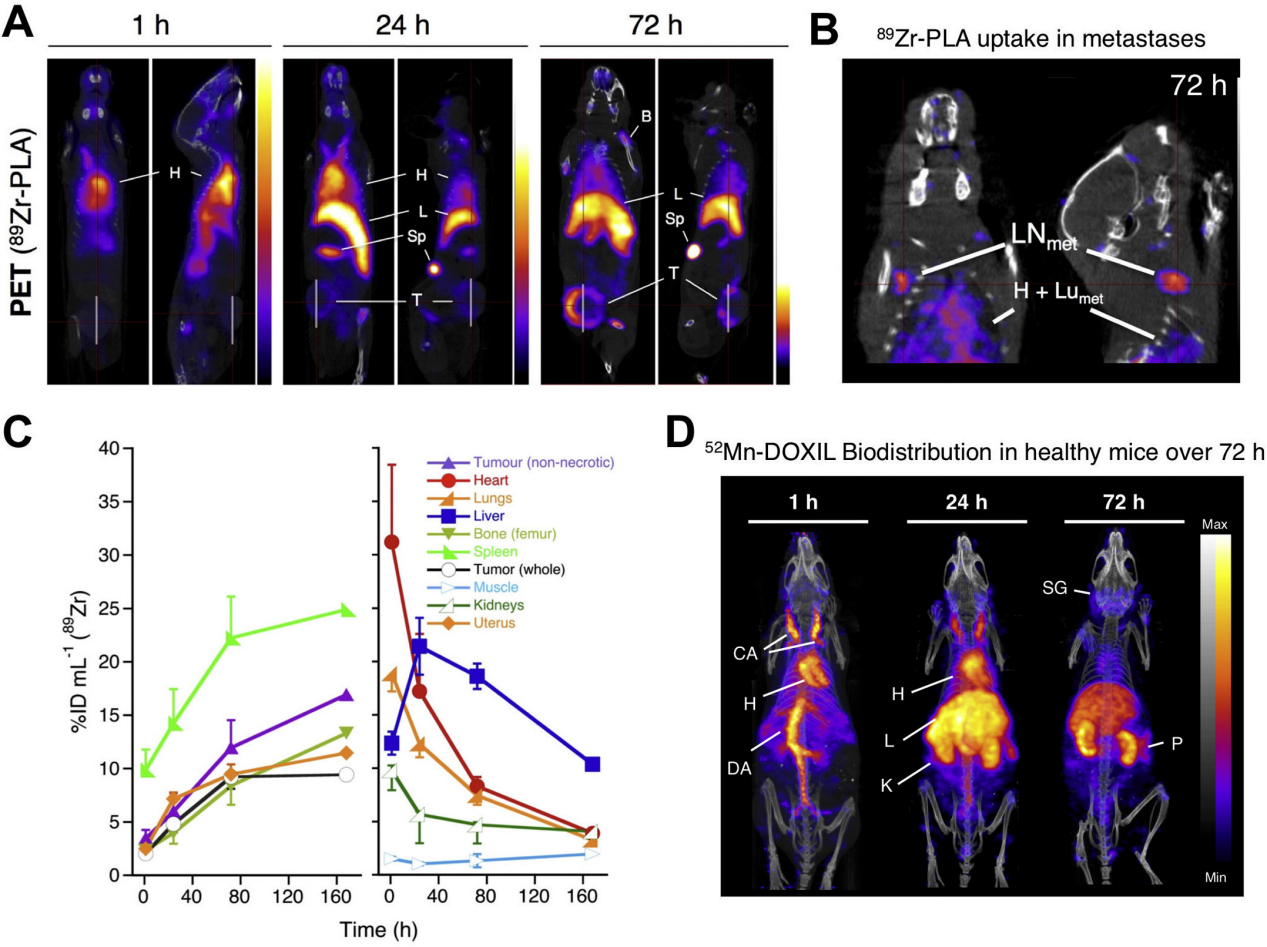
Ionophore-drug binding radiolabelling of liposomes. **(A)** Coronal and sagittal PET-CT images in the 3E.Δ.NT/NSG mouse model of metastatic breast cancer [143]. Images are centred at the tumours of the same animal from 1 h to 72 h after injection of ^89^Zr-PLA showing the increasing uptake over time in the primary tumour (T), spleen (Sp), liver (L) and bone (B), and decreasing uptake in blood pool/heart (H); (**B**) Coronal and sagittal PET-CT images centred at the LN_met_ of same animal from A at 72 h after injection of ^89^Zr-PLA, showing uptake of ^89^Zr-PLA in metastatic lymph node (LN_met_) and lungs (Lu_met_); (**C**) Time-activity curves (^89^Zr-PLA) from the study shown in A. (**D**) *In vivo* PET-CT imaging (MIPs) in a healthy B6CBAF1 mouse injected with [^52^Mn]Mn-DOXIL at 1, 24 and 72 h post-injection, and showing increasing uptake in kidneys/pancreas and salivary glands after 24 h, characteristic of free manganese, and thus suggesting Doxil cargo release (see Fig. 2). CA = carotid arteries; h = heart; DA = descending aorta; K = kidneys; SG = salivary glands; P = pancreas. Adapted with permission from Edmonds et al. [143], Copyright 2016 ACS.

The *in vivo* stability of the ^89^Zr-labelled PLA was demonstrated by the tracking of the liposomes within a metastatic breast cancer model, showing EPR-driven uptake in primary tumour and metastatic organs (lymph nodes and lungs) (Fig. 7A,B) [143]. The long-circulating properties of the liposomes were confirmed by a decrease in heart uptake over 72 h, which was contrasted with an increase in spleen uptake (Fig. 7C). Similarly, ^52^Mn-labelled Doxil® was shown to be stable in the blood pool for up to 24 h, however, imaging 72 h after administration and *ex vivo* biodistribution showed a profile similar to that of non-chelated ^52^Mn with high uptake in the pancreas, salivary glands and kidneys observed (Fig. 7D) [144]. These results suggest that following uptake in the reticulo-endothelial system (RES), the subsequent destruction of the liposomes led to the release of the drug cargo and the radionuclide. Indeed, when using ^89^Zr-PLA, uptake in the femur due to release of ‘free ^89^Zr’ was observed 72 h after administration (Fig. 7A,B). This release of the radionuclide demonstrates the need for caution when analysing images of radiolabelled nanomedicines. In particular, radioactive isotopes of endogenous metals, such as ^52^Mn and ^64^Cu, may be more susceptible to trafficking out of the tissues and into the bloodstream, resulting in secondary uptake in other organs. Specifically, in the case of ^64^Cu and ^52^Mn it may be difficult to separate free radiometal distribution from that of liposomal uptake in the liver and even in tumours (Fig. 2) [28,31]. This is less of an issue when labelling with ^89^Zr (a non-endogenous metal), which almost exclusively shows uptake in the bone [30].

#### 3.2.4 Remote loading

Finally, the labelling of pre-formed liposomes can also be achieved by the remote loading of metal complexes or radiopharmaceuticals inside the liposomal core (Fig. 4B). The first example of this was reported by Rudolph and collaborators who used ^99m^Tc-labelled hexamethylpropyleneamine oxime (HMPAO, Fig. 5E) to radiolabel pre-formed liposomes encapsulating albumin or haemoglobin along with glutathione [148,149]. It was found that glutathione was necessary to allow >90% LE, whereas liposomes encapsulated solely with albumin or haemoglobin had 11% and 19% LE, respectively. The authors demonstrated that the complex was trapped inside the aqueous liposome core, where it was postulated the complex would undergo reduction by interaction with glutathione, allowing trapping of the agent. This interaction had previously been proposed as the mechanism for the trapping of ^99m^Tc-HMPAO in the brain [150]. Cao et al. reported a similar method using ^99m^Tc-labelled diisopropyl iminodiacetic acid (^99m^Tc-DISIDA, Fig. 5E), again showing that liposomes containing glutathione resulted in higher uptake in the aqueous core [151]. Laverman et al. later compared HMPAO labelling with the use of HYNIC bound to the liposome surface [46]. Whilst there was no difference in serum stability after 48 h, the surface-labelled liposomes showed higher stability after incubation with DTPA, cysteine or glutathione. *In vivo* tracking showed that kidney uptake was 3-fold higher after 24 h for HMPAO-labelled liposomes, suggesting lower stability as ^99m^Tc-HMPAO is known to be renally excreted. However, it was also noted by the authors that the radiolabelled HYNIC-phospholipids would likely accumulate in the liver after degradation. This may make it difficult to elucidate liposomal signal in the liver, whereas in the case of HMPAO liposomes, renal uptake would avoid this issue. While both methods are simple, the need for modification of the liposomes, either by encapsulating glutathione or integrating HYNIC onto the surface, is a limitation of these methods for labelling nanomedicines.

Bao and collaborators developed an alternative remote-loading method using the chelator *N,N*-bis(2-mercaptoethyl)-*N’,N’*-diethyl-ethylenediamine (BMEDA, Fig. 5E) for ^99m^Tc [152,153], and later ^186^Re [154]. The neutrally charged complex allowed uptake into the aqueous liposomal core where it is protonated and becomes trapped in a more hydrophilic form. Initially it was shown that uptake of the ^99m^Tc complex into liposomes containing glutathione was moderate (*ca.* 37% LE), but with increased stability (>80%) in serum up to 72 h compared to empty liposomes (<35% stability) [152]. However, a subsequent study showed that the presence of glutathione resulted in lower stability compared to liposomes simply loaded with ammonium sulfate or citrate, irrespective of surface charge. In all cases, an improved LE was observed ranging from 66 to 84% [153]. The main advantage of this method is the ability to label preformulated liposomal nanomedicines without modification, as demonstrated by the use of this method for labelling and tracking of Doxil® both with ^99m^Tc- [155] and ^186^Re- BMEDA [156]. This technique was later used by other groups for loading the therapeutic radionuclide ^188^Re into liposomes to form a theranostic platform [[157], [158], [159], [160]].

The limitations in the use of a relatively short-lived radionuclide, as well as those with using SPECT, were eventually overcome by Lee et al. who developed a ^64^Cu complex capable of labelling liposome nanomedicines without modification [[161], [162], [163], [164]]. The ^64^Cu complex of diacetyl 4,4′-bis(3-(*N*,*N*-diethylamino)propyl)thiosemicarbazone (4-DEAP-ATSC, Fig. 5E) could be formed in just 1 min at room temperature with >94% RCY. ^64^Cu-4-DEAP-ATSC allowed >90% LE after 10 min at 65°C of two formulations of liposomal doxorubicin [161], as well as empty liposomes [162,164], indicating the labelling/trapping was not dependent on the presence of a encapsulated drug. Similarly to BMEDA, the neutral lipophilic complex becomes doubly protonated and charged, allowing it to be trapped in its hydrophilic form. The radiolabelled doxorubicin formulations showed high *in vitro* stability in serum (>99% after 48 h) and *in vivo* stability at 24 h. *Ex vivo* biodistribution showed higher splenic uptake of radiolabelled targeted doxorubicin liposomes compared to ^64^Cu-complex 24 h after administration, however, both radiopharmaceuticals showed similar uptake in the liver and kidneys. This is likely due to release of free ^64^Cu, as it is known that copper-bisthiosemicarbazone complexes are not stable *in viv*o [165]. Thus, any ^64^Cu-4-DEAP-ATSC released from the liposome will decompose and release free ^64^Cu. This is consistent with the observation from the authors showing that both the ^64^Cu-4-DEAP-ATSC and ‘free ^64^Cu’ had similar pharmacokinetics. Thus, this distribution of copper after release of the complex due to destruction of the liposomes should be taken into account, especially as ^64^Cu in its free form or as part of a bisthiosemicarbazone complex, is known to accumulate in tumours at similar levels (Fig. 2) [165,166]**.** Indeed the authors showed *ca.* 3 %ID/g tumour uptake of ^64^Cu-4-DEAP-ATSC 24 h after administration [161].

Most recently, Engudar et al. reported a novel radiolabelling method using a radioiodinated compound, amino diatrizoic acid (ADA, Fig. 5E), which could be loaded into liposomes using a transmembrane pH gradient [167]. ^125^I-ADA and ^124^I-ADA could be prepared with radiochemical yields of up to 64% and 55%, respectively, with radiochemical purities >90%, albeit after a lengthy purification process. The agents could be incorporated into liposomes after increasing the external pH to 7, the unprotonated compound then passively crossing the bilayer to become protonated and trapped inside. The maximum LE achieved labelling with ^124^I-ADA was 86% after 6 h of stirring at 55°C, though >70% LE could be achieved after just 2 h, with labelled liposomes shown to be 98% stable in HEPES buffer after 168 h. ^124^I-labelled liposomes showed long circulating properties with a blood t_1//2_ = 19.7 h compared to ^124^I-ADA which was rapidly cleared after just a few hours. Low deiodination of the liposomes occurred, evidenced by just 1 %ID/g of the radioactivity in thyroid present after 72 h. However, the authors note that free ^124^I-ADA may be released after uptake in organs and tumours, after which it will be rapidly cleared. This may lead to a biasing of the blood half-life and also organ uptake over time [167].

In summary, the radiolabelling of a liposomal nanomedicine should not be treated as a ‘black box’. Every aspect of radiolabelling, from the radionuclide and chelator choice to the location of the radiolabel incorporation, can have effects on the pharmacokinetics and biodistribution of the nanomedicine observed *in vivo*. As a result, the radiolabelling method should be carefully chosen based on the purpose of the study being performed. The biodistribution of the ‘free’ radionuclides, radiometal complexes/radiolabelled compounds and radiometal complex-lipid conjugates/amphiphilic probes should also be considered – depending on the method used – as each may be released following destruction of the liposomes and will potentially complicate the analysis of the images.

## 4 Applications of radiolabelled liposomes

As mentioned previously, radiolabelled liposomes have had numerous applications, covering *in vitro*, preclinical and clinical studies. As we recently reviewed clinical studies using radiolabelled nanomedicines [9], this section will mostly focus on preclinical studies, with an emphasis on developments in the last 10-15 years. Most studies are in the field of oncology, however radiolabelled liposomes have also been used in inflammation, infection, cardiovascular diseases, dermatology and other diseases.

### 4.1 Formulation

The pharmacokinetic properties of liposomes can be modified by changes in their size, chemical composition of the lipid bilayer, surface charge and other surface modifications. This extensive area of research has been summarised in recent reviews [1,168] and will only be briefly covered in this article. Radiolabelling liposomes of different compositions is a convenient way to assess the effect of individual modifications on their whole-body distribution and has been used since the early days of liposomal development [35,169]. For example, Richardson et al. showed greater uptake in rat tumours when using negatively charged liposomes [35]. The high uptake of liposomes by the RES has been known since the early days of liposome research. As a consequence, many strategies were investigated to reduce RES uptake and increase circulation times, using various radiolabelling methods to investigate the effect of RES blockade [170], liposome size, charge, dose, and lipid composition [105,118,[171], [172], [173]], mostly in health animals. The first study to report an increased uptake of a long-circulating liposomes in tumours was published by Gabizon and Papahadjopoulos [174].

With the increasing availability of radiometals for biomedical research, it is perhaps surprising that so many studies still rely on ^99m^Tc and planar scintigraphy. The study by Helbok et al. described in Section 3.1 took the approach of comparing instead different radionuclides for a same lipid formulation [58]. A formulation flexible enough to accommodate different radionuclides would give the user the flexibility to choose the most appropriate radiometal for the intended application. Here the formulation including DTPA was found acceptable (stable and with high specific activity) for ^99m^Tc, ^111^In and ^68^Ga, but sub-optimal for ^177^Lu. Beyond the proof of feasibility, however, a short-lived radionuclide such as ^68^Ga is not an ideal candidate for imaging formulations with long circulation times, and longer-lived PET radionuclides should be preferred. Bo et al. used ^89^Zr to image liposomes made from cancer cell membranes rather than synthetic lipids, with good stability of the labelling method over 72 h demonstrated by the low uptake of ^89^Zr in the bones [82]. This could be a useful approach to investigate whether the type of cancer cell from which the liposomes are made affects their distribution. Another option for increased flexibility is to take advantage of nuclides with several radioisotopes. For example, the formulation by Engudar et al. mentioned previously in Section 3.2.4, can be radiolabelled with ^124^I for PET imaging, ^125^I for SPECT imaging and Auger therapy, or ^131^I for beta therapy [167].

Sou et al. have studied drug delivery to the bone marrow by modifying the surface of liposomes with an anionic lipid ester [175,176]. The spatial resolution of nuclear imaging does not always allow easy differentiation between uptake in the bone and bone marrow in rodents, requiring the use of larger animals (*e.g.* rabbits) and independent confirmation of uptake, at least for initial studies. Here, dual fluorescent labelling of both the lipid membrane and aqueous compartment proved the integrity of the liposomes inside the bone marrow, and transmission electron microscopy showed the intracellular distribution within bone marrow macrophages [175]. Such detailed investigations are particularly welcome and show that a further challenge lies in demonstrating whether the encapsulated cargo can reach targets located outside endosomal vesicles. Nonetheless, Lee et al. labelled bone-marrow targeting liposomes with ^64^Cu and were able to observe PET signal originating from the bone marrow in mice femurs [74]. A combination of small size, negatively charged surface and reduced PEG load resulted in an increased bone marrow uptake compared to Doxil®-like liposomes. Jestin, Mougin-Degraef and collaborators developed lipid nanocapsule formulations that could be radiolabelled with multiple radionuclides (^99m^Tc, ^111^In, ^125^I, ^131^I) [55,56,177]. Although these systems were intended as vehicles for radionuclide therapy, using for example ^90^Y or ^211^At, dual labelling of the membrane lipids and encapsulated contents is a useful way to assess the integrity of the nanomedicine formulation after delivery. In this case, the stability of the formulation in blood followed by urinary elimination of ^125^I showed the disintegration of the carrier after uptake in the liver and spleen [56]. This aspect is often overlooked and simply assumed from the appearance of biological effects of the cargo. An example of this approach was recently given by Lamichhane et al., who encapsulated an ^18^F-labelled derivative of carboplatin into liposomes surface-labelled with ^111^In by SPECT to directly observe the in *vivo s*tability of the formulation. Similarly, Medina et al. radiolabelled an EGFR inhibitor with ^124^I and encapsulated it inside ^111^In-labelled liposomes [109]. Clear differences in the biodistributions of the radionuclides were noted after 24 h, indicating the release of the drug from the liposomes. There are nonetheless limitations to this approach. While covalent radiolabelling of a molecule offers unambiguous determination of its location, as opposed to co-encapsulation of a radionuclide which is then assumed to distribute similarly to the drug, not all drugs can be radiolabelled this way and it should still be determined that the therapeutic drug and its radiolabelled derivative have similar pharmacokinetics. Furthermore, the use of ^18^F will only provide stability information in the first few hours after administration. It is not an ideal radionuclide to image a drug with an elimination half-life of approximately 6 h, particularly if it is encapsulated in a long-circulating carrier. This approach also requires the liposomes to be prepared extemporaneously, after the ^18^F-labelling step. The use of ^124^I partly solves this problem but has its own complications since radioiodine-labelled molecules are prone to deiodination *in vivo,* meaning that part of the signal may no longer originate from the actual drug but from the released radionuclide. This additional complexity may limit the applicability of the dual-labelling approach to a preclinical setting.

The versatility of liposomes as imaging agents can be increased by multimodal approaches. Certain drugs, such as doxorubicin, are fluorescent and therefore radiolabelling liposomal formulations of these molecules will result in inherently bi-modal imaging tools. Optical and/or magnetic resonance imaging (MRI) capabilities can be incorporated as well. For example, liposomes containing DOTA-conjugated lipids were labelled with Gd^3+^ for MRI and ^64^Cu or ^111^In for nuclear imaging, as well as fluorescein- or near-infrared dye-conjugated lipids for optical imaging [72,178], and could additionally be remote-loaded with ^99m^Tc and doxorubicin. Each imaging modality showed a good retention of the formulation for 24 h after intratumoural administration [178]. Notably, Paoli et al. loaded liposomes containing ^18^F- or ^64^Cu-labelled lipids with fluorescent dyes to study the effect on drug release of various lipid compositions [66]. This study illustrates the benefit of labelling the encapsulated cargo: although increased drug release in one formulation could be deduced from the appearance of PET signal in the bladder (resulting from lipid metabolism), the increased optical signal was far greater and showed a much broader distribution of the released dye. To obtain meaningful results from imaging studies, it is therefore crucial to specifically (radio)label the constituent of interest, *i.e.* a liposomal membrane component or the cargo. The light-emitting properties of certain radionuclides have also been exploited to provide multimodal imaging: Kim et al. radiolabelled liposomes with ^124^I [179], which emits Cerenkov radiation and is thus detectable with luminescence imaging systems. The depth penetration issue of Cerenkov radiation was apparent from the absence of bladder signal in the optical scans, although this might be solved by re-positioning the animal for a second scan, which is easily feasible with little consequences because of the short acquisition times (a few minutes) required for luminescence imaging. Liposomal degradation was apparent from the signal emanating from the thyroid after 24 h, both by optical and PET imaging.

Most studies of radiolabelled liposomes provide data on the *in vitro* stability of the formulation, but few have investigated in depth the *in vivo* release of radiolabelled molecules from liposomes. To establish an *in vitro-in vivo* correlation (IVIVC), Hühn et al. encapsulated [^18^F]FDG into liposomes, injected them intraperitoneally and then used PET to measure the uptake of [^18^F]FDG in the brain [180], where this tracer naturally accumulates after reaching the circulation. Thus, appearance of signal in the brain could only come from radiotracer release from the liposomes. Similar approaches could be envisaged with other radionuclides, using for example the uptake of radioiodine in the thyroid or ^89^Zr in the bone to determine liposomal stability. The development of IVIVCs for liposomal formulations would be highly beneficial for their clinical development, giving much more power to the routinely performed *in vitro* stability tests, and nuclear imaging can certainly play an important role in progressing these drugs towards the clinic. A recent and noteworthy example of *in vivo* analysis of drug release is provided by Mukai et al., who combined PET imaging of ^64^Cu-labelled oligonucleotides with LC-MS/MS analysis of tissue samples [181]. Mass spectrometry showed that the oligonucleotides were so rapidly degraded *in vivo* that they could only be detected intact in the kidneys, the PET signal therefore representing mostly metabolites and/or unchelated ^64^Cu. In contrast, encapsulating the oligonucleotides in liposomes preserved them from degradation. The comparison of PET and LC-MS/MS data showed that release of the oligonucleotides from the liposomes and subsequent degradation occurred much faster in the liver than in other tissues. With the liposomal oligonucleotides, the PET signal in the tumour increased over 48 h whereas the amount of intact oligonucleotide determined by mass spectrometry peaked around 24 h. Consequently, the PET signal in the organ of interest is a measure of the cumulative drug delivery, but the actual fate of the drug is more accurately measured by other means. Considering the relatively wide availability of LC-MS/MS and the possibility of preserving samples for off-site analysis, this is a technique that we feel should be far more frequently used in conjunction with nuclear imaging, certainly at the preclinical development stage.

### 4.2 Oncology

#### 4.2.1 Diagnosis

Although liposomes were initially studied mostly for their use as drug carriers [1], the use of radiolabelled liposomes in oncology started briefly after their invention when Gregoriadis et al. first administered ^131^I-loaded liposomes in three cancer patients and observed a much higher uptake in cancerous kidney tissue compared to healthy tissue [103]. The first imaging studies were performed a few years later by Richardson et al., using ^99m^Tc, and already hinted at potential differences between animal tumour models and human tumours, and between patients [36,38]. Further studies helped to establish the safety of liposomes in patients and showed that radiolabelled liposomes could identify unsuspected tumours and thus serve as diagnostic agents [[182], [183], [184], [185], [186], [187], [188]]. With the gradual shift towards the use of long-circulating, PEG-coated liposomes, the variability of the EPR effect in humans become even more apparent, as illustrated in the landmark study by Harrington et al. [189]. A drawback of using radiolabelled liposomes for tumour imaging is their slow accumulation in tumours, and many of these clinical studies found that diagnostic accuracy was improved when imaging was delayed by 24-48 h after administration [183,184,190]. From a clinical perspective, this complicates logistics by requiring the patient to attend at least 2 visits, and radiolabelled liposomes appear not to have been used much further in a purely diagnostic setting. Considering the established clinical use of [^18^F]FDG and other PET radiotracers, with excellent performance in cancer diagnosis and staging, it seems unlikely that liposomes will be used for tumour detection. In recent preclinical studies, Wong, Mahakian, Rygh and colleagues provided useful comparisons of ^64^Cu-labelled liposomes, ^64^Cu-labelled albumin and [^18^F]FDG, showing that the liposomes were superior to [^18^F]FDG in detecting small tumours and revealing heterogeneities within tumours, especially when imaging after 18-24 h [[67], [68], [69]]. ^64^Cu-labelled liposomes could be a useful alternative to [^18^F]FDG for tumours located close to organs with constitutively high [^18^F]FDG uptake such as the brain, the heart, and the kidneys/bladder. This comes at the expense of high background signal of radiolabelled liposomes in the abdominal region and the requirement for delayed imaging, which are clear limitations of the technique and would still favour [^18^F]FDG as a general radiotracer for tumour detection. An additional consideration is that using a radionuclide with a longer half-life prolongs the exposure of the patient and may result in a higher absorbed radiation dose. Interestingly, ^64^Cu-labelled albumin proved better than liposomes for imaging increases in vascular permeability during tumour progression, as uptake in tumours increased more gradually than that of liposomes [67], and further suggests that radiolabelled liposomes are probably better for monitoring liposomal drug delivery than for tumour detection. Using radiolabelled liposomes in conjunction with small-molecule radiotracers could be a strategy to interrogate both the vascular permeability and metabolic status of tumours. The use of radiolabelled liposomes for the prediction of treatment response and patient stratification, where tumour heterogeneity is particularly important, is discussed further in this review (Section 4.6).

A common issue to both radiolabelled liposomes and [^18^F]FDG is that they can fail to differentiate tumours from sterile inflammation or infectious foci. A few recent articles have explored the possibility of using radiolabelled liposomes containing diagnostic agents with a higher specificity for tumours. One such approach is to use radiolabelled antisense oligonucleotides that recognise mRNA sequences coding for proteins involved in tumourigenic processes [191]. The high specificity of the probes offers the potential to achieve high target-to-background ratios, but the bioavailability of nucleic acids is poor and carrier systems, including liposomes, are generally required for effective delivery [192]. Fu et al. incorporated ^99m^Tc-labelled antisense oligonucleotides directed against the MDM2 oncogene into liposomes (Lipofectamine®) and observed a 3-fold increase in tumour uptake compared to a mismatched oligonucleotide [193]. Some accumulation of the mismatched probe in the tumours can be seen in the SPECT images, likely due to EPR-mediated uptake. Using the same liposomal carrier, Liu et al. imaged an antisense nucleotide directed at telomerase reverse transcriptase, based on the fact that many tumour cells maintain their proliferative ability by sustaining telomerase [194]. In this study, although the tumour uptake of the antisense probe was far higher than the sense probe, the imaging study revealed no difference between the liposome-encapsulated and non-encapsulated formulations, in direct contradiction of the observed *in vitro* effectiveness of this strategy. In the absence of full biodistribution data, it is not clear whether the liposomal formulation led to differences in biodistribution of the oligonucleotide probe. Nonetheless, these two studies illustrate the benefit of imaging the encapsulated drug, whenever possible, rather than the carrier. In the first study, it is not determined whether liposomal encapsulation provides any benefit, and a non-liposomal control would have been warranted. In the second study, radiolabelling the liposomes on their surface or remote-loading a radionuclide that does not bind to the cargo would probably not have revealed the specific accumulation of the antisense oligonucleotide. Another approach is to coat liposomes with tumour-targeting peptides, directed for example against integrin α_V_β_3_, a protein overexpressed in many tumours [195]. Kang et al. radiolabelled α_V_β_3_-targeting liposomes with ^64^Cu to monitor tumour angiogenesis [73]. Despite the images showing a higher and faster uptake of ^64^Cu with the targeted liposomes compared to non-targeted liposomes, the intense accumulation in the liver did not compare favourably to an existing small-molecule, ^18^F-based radiotracer targeting the same protein.

#### 4.2.2 Drug delivery

Despite liposomes being extensively researched as drug carriers, very few studies of radiolabelled liposomes until the early 2000s included formulations containing an active pharmaceutical ingredient [123,196,197]. While it may seem easier to use ‘empty’ liposomes, *i.e.* not containing any cargo, co-encapsulating a drug and a radiotracer or encapsulating a radiolabelled drug allow direct correlation of uptake measurements with therapeutic efficacy and therefore provide more information. Two clinical studies by Koukourakis et al. illustrate this approach, where it was shown in patients with non-small-cell lung cancer that tumour uptake of ^99m^Tc-labelled liposomal doxorubicin (Doxil®) correlated with tumour vascularisation and appeared to correlate with tumour regression, although further data would have been required to prove the latter [198,199]. Repeated administration of the radiolabelled liposomes was performed and scintigraphy demonstrated high uptake in tumours both before and after radiotherapy [198], suggesting in hindsight that there was no significant involvement of the accelerated blood clearance (ABC) effect (see Section 4.6).

A major benefit of using nuclear imaging is the ability to quantify drug uptake and monitor disease status in organs not readily accessible by other means. For example, a number of small-molecule PET radiotracers are clinically available for brain tumour imaging, such as [^11^C]choline, [^18^F]FDG, [^18^F]FLT or [^18^F]FET, but liposomal formulations could potentially be used both for diagnosis/monitoring and drug delivery. Increased transport across the blood-brain barrier, a major challenge in drug development, was demonstrated with ^99m^Tc-labelled liposomes coated with transferrin [200,201]. ^18^F-labelled liposomes proved superior to [^18^F]FDG in a rat model of glioma, being capable of detecting very small tumours and showing lower background signal in the brain [92], although for imaging purposes the added value of an angiogenesis-targeting peptide compared to non-targeted PEGylated liposomes was less evident. In another study, incorporating an ^18^F-labelled derivative of dasatinib (a platelet-derived growth factor receptor inhibitor) into liposomes provided no benefit over the non-encapsulated drug, although it is worth noting these were non-PEGylated liposomes and can thus be expected to rapidly accumulate in the RES, and a PEG-based micellar formulation of the same radiotracer increased the amount of tracer reaching brain tumours [110]. One might speculate that higher accumulation in the brain could potentially be achieved with longer-circulating liposomes, in which case a different radionuclide would certainly be required for useful imaging.

Two recent studies by Patel et al. investigated drug delivery to the uterus for the treatment of endometriosis [202,203]. Raloxifene and leuprolide were directly radiolabelled with ^99m^Tc before encapsulation in liposomes for intravaginal administration. Both drugs have poor uterine bioavailability and significant side effects when administered parenterally. Scintigraphic imaging revealed much longer retention of the liposomal drugs in the uterus compared to the non-encapsulated drugs, and slow release was visible for leuprolide from the delayed accumulation in the kidneys. Although therapeutic efficacy was not assessed in these studies, the absence of signal from other organs suggests a low risk of side effects.

A strategy to increase liposomal retention in lymph nodes and in the peritoneum by using the avidin-biotin system has been investigated using nuclear imaging [[204], [205], [206], [207]]. The underlying concept is that the administration of avidin can induce the aggregation of biotin-conjugated liposomes. Another approach to enable the prolonged release of liposomes from a reservoir is to embed them in a hydrogel, which can be delivered locally. Alinaghi et al. added ^99m^Tc-labelled liposomes to a chitosan-glycerophosphate hydrogel which was then administered intraperitoneally [208]. The hydrogel formulation significantly increased the amount of ^99m^Tc in the blood and peritoneal cavity and reduced uptake in the liver. Beyond the advantages of lower systemic exposure and higher local concentration conferred by the intraperitoneal or subcutaneous delivery modes, radiolabelling allowed both longitudinal studies and uptake measurements in organs not easily accessible by necropsy.

The trafficking of liposomes to tumours can be increased by local hyperthermia, which increases vascular permeability. Nuclear imaging has been used to demonstrate this with ^99m^Tc-labelled liposomes after heating with a catheter [209], microwave irradiation [210] or radiofrequency ablation [211]. Scintigraphic and SPECT measurements of ^99m^Tc accumulation in the tumours correlated well with HPLC measurements of intratumoural doxorubicin, although the actual release of the drug from the liposomes was not studied. Similarly, Oerlemans et al. used MR-guided focused ultrasound to release ^99m^Tc from liposomes [212], with confirmation of release from the liposomes provided by co-encapsulating fluorescein, whose fluorescence exhibits self-quenching at high intraliposomal concentrations and increases due to dilution upon release. Encapsulating ammonium bicarbonate into liposomes provided a thermally sensitive yet more stable formulation than the lysolipid-based ThermoDox® [213]. Here, the lower stability of ThermoDox® was observed by SPECT with an increased kidney uptake of ^99m^Tc at early time points, which resulted in lower tumour uptake and therapeutic efficacy.

Perche et al. prepared electrostatic complexes of radiolabelled nucleic acids and liposomes, called lipoplexes, to develop a dendritic cell vaccine [214]. The lipoplexes were mannosylated to increase uptake by dendritic cells, although this point was proven by *ex vivo* fluorescence measurements. Since the distribution patterns were similar between mannosylated and non-mannosylated lipoplexes, the main lesson from the radiolabelling was the absence of accumulation of the nucleic acids in the lungs, which can occur after aggregation of the lipoplexes.

Bisphosphonates are small molecules with high affinity for bone minerals and are used to combat osteolytic diseases due to their anti-osteoclastic action. Liposomal encapsulation of bisphosphonates has been explored as a way of exploiting their anti-cancer properties in other tissues. Hodgins et al. radiolabelled liposomal alendronate with ^111^In – using DSPE-DTPA – in a mouse model of melanoma, in combination with γδ-T cell therapy [215,216]. It is worth noting that the chelating properties of bisphosphonates enable the direct radiolabelling of these compounds [143,145] and it might be preferable to use this approach to image the drug rather than the liposomal carrier. *In vitro* results showing improved efficacy of integrin αvβ6-targeting liposomes compared to non-targeted liposomes were not replicated *in vivo*, where imaging showed no difference in tumour uptake or therapeutic efficacy. The combination with γδ-T cells demonstrated strong anti-tumoural activity, suggesting the additional complexity of targeting liposomes may not be required for this type of cell therapy.

**Fig. 8:**
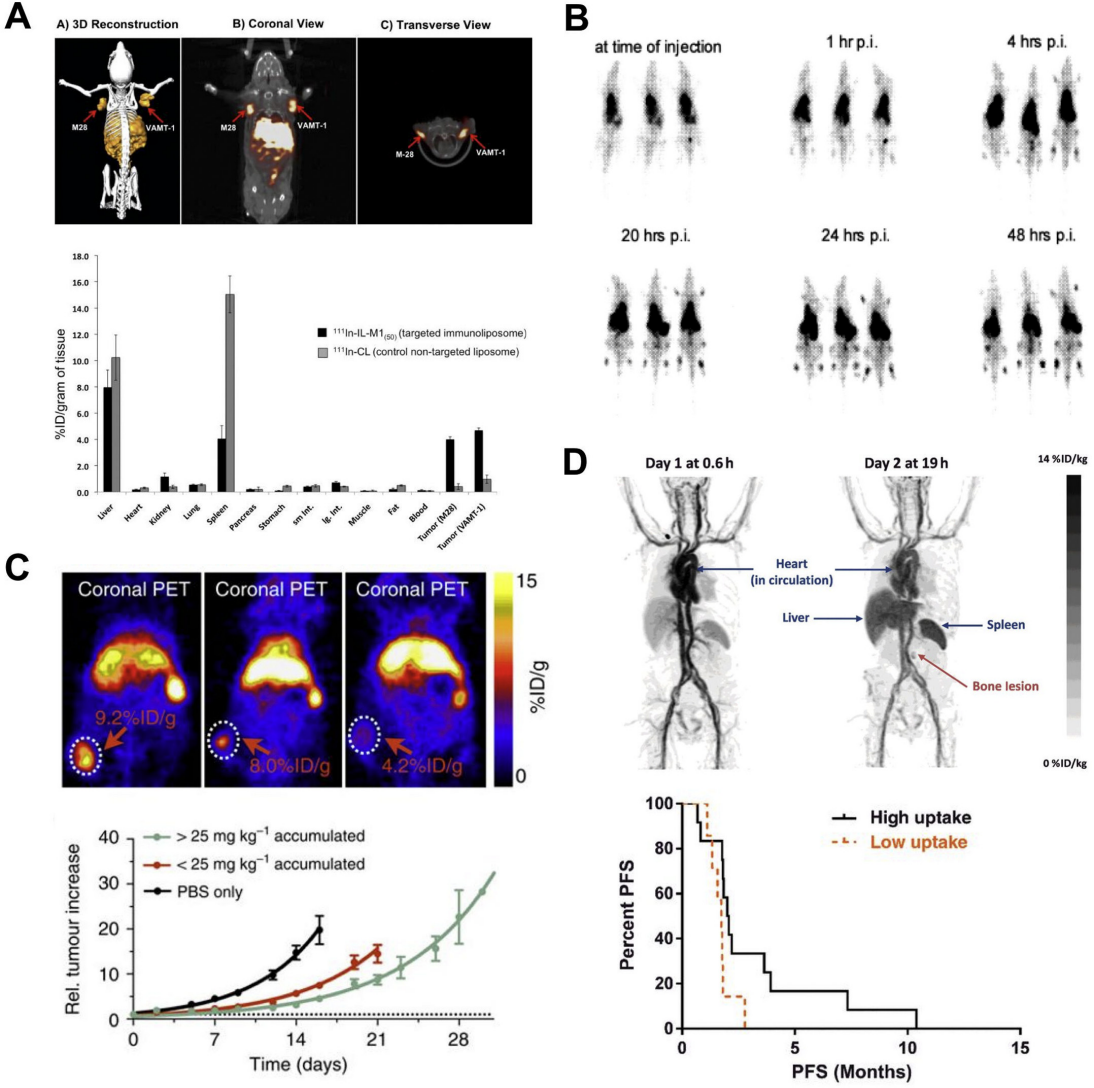
Preclinical and clinical examples where nuclear imaging has been used to answer specific liposomal therapy questions. **(A)** Introduction of a single chain antibody (scFv) tumour-targeting group into liposomes (immunoliposomes – ILs) improves tumour uptake. (Top) SPECT/CT images of ^111^In-ILs 24 h after injection showing uptake of in both epithelioid (M28) and sarcomatoid (VAMT-1) mesothelioma tumours. (Bottom) ILs show higher tumour uptake compared to non-targeted liposomes (CLs). Adapted with permission from Iyer et al. [59], Copyright 2011 Elsevier. **(B)** Glucocorticoid-loaded PEGylated liposomes radiolabelled with ^111^In demonstrate high-EPR mediated uptake over time in inflamed joints in a model of rheumatoid arthritis. Adapted with permission from Metselaar et al. [129], Copyright 2003 John Wiley and Sons. **(C)** PEGylated liposomes radiolabelled with ^89^Zr allow non-invasive quantification of therapeutic liposome (*e.g.* Doxil®) tumour uptake using PET (top) and allow therapeutic efficacy prediction based on PET signal concentration at the tumour (bottom). Adapted from Pérez-Medina et al. [79] under CC-BY license [275]. **(D) (**Top) Maximum intensity projection PET images of a patient with HER2-positive breast cancer injected with ^64^Cu-MM-302. As expected from long circulating liposomes at 0.6 h post injection most activity is in circulation, and only from day 2 is significant uptake in RES organs and tumour lesions evident. (Bottom) A correlation between ^64^Cu-MM-302 tumour uptake levels (high or low) and patient progression-free survival (PFS) is seen, although not statistically significant due to low numbers. Adapted by permission from the American Association for Cancer Research: Lee et al. [163].

On the other hand, ^111^In-labelled liposomes decorated with an internalising scFv showed a fourfold increase in tumour uptake compared to untargeted liposomes and very high tumour-to-blood and tumour-to-muscle ratios (Fig. 8A) [59]. This was mirrored by a lower uptake in the liver and spleen. The slightly higher kidney uptake with the targeted formulation may suggest a competing effect between the scFv and ^111^In-DTPA groups on the liposomal surface leading to the release of ^111^In, or some liposomal degradation upon internalisation. It would be valuable to determine whether this increased uptake can deliver improvements in therapeutic efficacy. Interestingly, this study determined the optimal amount of targeting moiety to promote internalisation. As a counterpoint, Christensen et al. recently showed that ^64^Cu-labelled folate-targeting liposomes had lower tumour uptake than non-targeting liposomes *in vivo* despite numerous reports of increased uptake *in vitro*, the authors suggesting that the targeted receptor was not accessible to the liposomes and that the EPR effect was predominant [217]. Aside from the differences in tumour models, which may have varying degrees of vascularisation and EPR effect [218], these discrepancies could also be due to the specific targeting moieties, the extent to which they promote internalisation and their density on the liposomal surface. These studies using targeted liposomes highlight the value of nuclear imaging: the question of whether the targeting of these nanoparticles is effective can be rapidly answered, usually within 24 h. The need to wait for a biological response (*e.g.* changes in tumour size), which typically takes several days and may require multiple administrations, is also removed. They also highlight the importance of including non-targeting formulations as controls for *in vivo* experiments, since *in vitro* results cannot be assumed to be predictive.

#### 4.2.3 Radionuclide therapy

Liposomes can be labelled with radionuclides suitable for radionuclide therapy, such as the alpha-emitters ^166^Ho, ^225^Ac and ^213^Bi [63,102,135,136,219], beta-emitters ^90^Y, ^131^I, ^159^Gd, ^177^Lu [62,101,121,131,220] or ^186^Re/^188^Re [154,157,159,160,[221], [222], [223], [224], [225]], and Auger electron-emitters ^111^In or ^125^I [177,[226], [227], [228]]. The advantages and disadvantages of using each type of particle in anticancer radiotherapy are beyond the scope of this article but have been reviewed recently elsewhere [229,230]. There are nonetheless practical implications for liposomal formulations. The very short range of Auger electrons (a few nm) means these radionuclides need to be delivered as close as possible to the cell nucleus to cause damage. Alpha- and beta-emitters have much larger energy deposition volumes and therefore liposomal escape from endosomal vesicles and release of the radionuclide from the liposome are not required for therapeutic efficacy, with the downside of an increased radiation burden to neighbouring healthy cells. Several of the above radionuclides also emit gamma rays, enabling simultaneous imaging and treatment (see Table 1). Fondell et al. illustrated this concept with a liposomal formulation incorporating a ^125^I-labelled daunorubicin analogue, in which the damage to tumour cells was driven by the emissions from ^125^I rather than the DNA-intercalating properties of the drug [226]. Another example is given in the studies by Lin, Chow and colleagues using formulations combining ^111^In and the chemotherapeutic drug vinorelbin [227,228]. This work highlights the importance of choosing the appropriate models to study, as the therapeutic effect of liposomal vinorelbin was so pronounced that it was difficult to evaluate the added value of ^111^In as a radiotherapeutic agent beyond its use for imaging. In another study, Zavaleta et al. used the biotinylated liposome formulation for intraperitoneal delivery mentioned above [206] and substituted ^99m^Tc for ^186^Re, providing a radiotherapeutic system that also allowed imaging over 5 days [223]. This flexible labelling approach could be applied to personalized medicine by using the ^99m^Tc-labelled formulation to explore the distribution of the nanomedicine and following up with the ^186^Re-labelled version if the distribution is deemed favourable, as exemplified in a rat model of glioblastoma [222]. A similar strategy could be pursued with the ^64^Cu/^177^Lu pair of radionuclides described by Petersen et al. [220]. Here, PET imaging of ^64^Cu was proposed as a preliminary step before administration of radiotherapeutic ^177^Lu. Considering that the liposomal formulation led to high accumulation in the liver and spleen, a more detailed study would be welcome to assess whether sufficient amounts of ^177^Lu can be delivered to the tumour while minimising hepato-splenic toxicity. Liposomes loaded with ^188^Re were studied in several tumour models and appeared well-tolerated, with accumulation in tumours observable by SPECT, but could not eradicate established tumours at tolerated doses [[157], [158], [159]], although the co-encapsulation of doxorubicin increased therapeutic efficacy [231]. To counter potential toxicity issues and increase the therapeutic efficacy, local delivery to the tumour has been explored as an alternative to the intravenous route. Studies using ^186^Re-loaded liposomes demonstrated therapeutic efficacy in a tumour resection model, particularly for cationic liposomes compared to neutral liposomes due to increased retention at the site of administration [221,232]. Imaging demonstrated much lower uptake in the liver than would be expected after systemic administration. Here, radionuclide therapy is envisaged as an adjunct to surgical resection, to kill residual tumour cells. Together, this body of research suggests that radionuclide therapy delivered by liposomes is probably best used in combination with other treatment modalities rather than as an alternative to chemo- or radiotherapy. Finally, formulations combining paramagnetic metals and therapeutic radionuclides have potential for multimodal imaging, using for example gadolinium and ^166^Ho or ^159^Gd, although studies do not appear to have progressed beyond the *in vitro* stage [63,101].

### 4.3 Infection, inflammation

Nuclear imaging of infections was historically performed with ^67^Ga-citrate, ^111^In-immunoglobulin G or white blood cells radiolabelled with ^111^In-oxine or ^99m^Tc-HMPAO. Due to the increased blood flow and vascular permeability, and in some cases increased phagocytic activity, liposomes also accumulate in inflamed tissue areas. This property has been exploited to deliver anti-inflammatory and antibiotic compounds [[233], [234], [235], [236], [237]]. The most used drug in this category is AmBisome® [238], a liposomal formulation of amphotericin B, although in this case the main benefit of the liposomal formulation is simply a reduction in toxicity [239]. Radiolabelled liposomes can also be used to identify sites of infection and inflammation [[240], [241], [242]], particularly as an easier alternative to the comparatively complex use of radiolabelled white blood cells. Liposomes also proved superior to ^67^Ga citrate in neutropenic rat models of bacterial and fungal infection [243], where the number of circulating leukocytes would be too low for radiolabelling, although nowadays [^18^F]FDG PET seems more indicated in such cases [244]. Andreopoulos et al. attempted to use ^99m^Tc-labelled liposomes as vehicles to radiolabel white blood cells in whole blood [245], to solve the problem of ^99m^Tc efflux from leukocytes after HMPAO-mediated labelling and to avoid the lengthy leukocyte isolation process. However, the radiolabelling efficiency was very low and the activity concentrated mainly in mononuclear cells, which are not the main responders to infections. One of the earliest studies of radiolabelled liposomes to detect infectious abscesses was that performed by Morgan et al., where negatively charged liposomes rapidly accumulated in abscesses caused by *Staphylococcus aureus* [246]. Orozco et al. observed a much higher accumulation of radiolabelled liposomes in the lungs of tuberculous mice compared to normal mice [247], which might have explained the higher efficacy of their liposomal formulation of rifampicin and isoniazid. The study by Bakker-Woudenberg et al. in a *Klebsiella pneumoniae* model is particularly interesting in that it showed a strong correlation between liposomal uptake in the lungs and the severity of infection, assessed both by the mass of the lungs and the number of bacteria present [248]. For example, Sikkink et al. have used liposomes labelled on their surface with ^99m^Tc to detect abdominal abscesses in a rat peritonitis model [48], showing good correlation between focal uptake of liposomes and the presence of intra-abdominal adhesion and potential for treatment monitoring. Based on the local acidification that occurs in inflamed tissues, Carmo et al. have used pH-sensitive liposomes labelled with ^99m^Tc to detect sterile inflammation foci [249], claiming faster and higher uptake than for non-pH-sensitive formulations [242,250]. This would enable faster and more robust detection of inflammation, although the study unfortunately did not include a direct comparison between formulations and the disease model was different (sterile inflammation *vs*. *S. aureus* osteomyelitis). Distinction between bone infection and sterile inflammation in later studies with ceftizoxime-encapsulating pH-sensitive liposomes was less evident, despite improved uptake in the bones with alendronate-coated liposomes [251,252]. More generally, this is a shortcoming of many studies using radiolabelled liposomes. It is not sufficient to claim that a formulation can detect foci of infection or inflammation. To help clinical adoption, studies should include additional experimental comparisons and make the case that radiolabelled liposomes are equivalent or better than currently used tracers, as exemplified in preclinical studies comparing liposomes with radiolabelled leukocytes or immunoglobulin G (IgG) [253,254]. A direct, within-subject comparison of ^99m^Tc-labelled liposomes and ^111^In-IgG was performed in 34 patients with suspected infection or inflammation. The liposomes showed marginally higher sensitivity, similar specificity and generally improved image quality over radiolabelled IgG [255]. The only other clinical trial of radiolabelled liposomes for infection was performed by Weers et al. with liposomal amikacin for inhalation (LAI) and showed prolonged retention of activity in the lungs, although it was only assessed in healthy volunteers [256]. LAI is still under clinical evaluation, and a recent trial reported inconclusive results in patient with pulmonary non-tuberculous mycobacterial disease [257]. Incorporating imaging in such studies would provide a better picture of drug distribution than blood sampling and potentially elucidate whether treatment failure is caused by inadequate drug distribution or bacterial resistance.

The first clinical reports of accumulation of radiolabelled liposomes in patients with rheumatoid arthritis were probably those by Morgan, Williams, O’Sullivan and colleagues [[258], [259], [260], [261]] in the late 1980s, already suggesting the use of such formulations to monitor phagocytic activity in the synovial tissue as better markers of disease status than the anatomical changes observed by X-ray imaging. Furthermore, Zalutsky et al. suggested using ^111^In-labelled liposomes for radiation synovectomy [262]. In veterinary medicine, Underwood et al. have shown that liposomes could detect laminitis in horses and highlighted potential systemic inflammation in this debilitating disease [263,264]. Türker et al. administered ^99m^Tc-labelled liposomes intra-articularly to study their retention [265]. Although the radiolabelling was not specific to any constituent of the liposomes, scintigraphy showed good retention of the radiotracer in the inflamed joints, and urinary excretion of released ^99m^Tc but not hepatosplenic uptake. This approach could be useful for anti-inflammatory drugs with hepatosplenic toxicity or rapidly degraded in the liver. Corvo et al. showed that subcutaneous administration of small-sized (< 150 nm) liposomes loaded with superoxide dismutase was equally effective as the intravenous route in reducing inflammation [100,130]. In another therapeutic study, Metselaar et al. observed that liposome encapsulation of prednisolone significantly increased the therapeutic activity compared to the non-liposomal drug, and imaging showed that this was due to the increased circulation time and accumulation of the drugs in the joints (Fig. 8B) [129]. This product is now in phase III clinical trials for rheumatoid arthritis treatment (NCT02534896). Liposomal prednisolone was recently evaluated in a clinical trial for atherosclerosis treatment, and the same group of authors commented that the apparent absence of therapeutic response may have been due to insufficient drug accumulation and that this uncertainty could have been solved by using non-invasive imaging such as radiolabelled liposomes [266].

Apart from arthritis, radiolabelled liposomes have also been used in models of abdominal inflammation (colitis, inflammatory bowel disease, Crohn’s disease) [49,267,268] and brain inflammation [269]. Notably, in a study by Awasthi et al., ^99m^Tc-labelled liposomes performed better than ^111^In-labelled leukocytes [268].

Saari et al. conducted as series of clinical studies in healthy volunteers and asthmatic patients using ^99m^Tc-labelled liposomes loaded with beclomethasone, a corticosteroid widely used as a non-liposomal aerosol in asthma management [[270], [271], [272], [273]]. Scintigraphy showed that the lung deposition of the liposomal formulation was not affected by concomitant treatment with a long-acting β2-agonist. Although the development of this liposomal formulation does not appear to have continued, these studies show that using nuclear imaging is an interesting option to evaluate interactions between drugs in combination therapies. More recently, Behr et al. imaged liposomal cyclosporine A in lung transplant recipients, showing good deposition in the lungs [274].

A common feature of the studies mentioned here is that the sensitivity of nuclear imaging for the detection of infectious and inflammatory foci is somewhat offset by the difficulty in distinguishing these two phenomena. There are therefore opportunities to develop targeting strategies to improve diagnostic accuracy and drug delivery.

### 4.4 Cardiovascular

Recently, Ogawa et al. have used ^111^In-labelled liposomes to image atherosclerotic plaque, exploiting the presence of infiltrated macrophages in vulnerable plaque [276]. The liposomes were coated with phosphatidylserine to mimic apoptotic cells and trigger phagocytosis by the macrophages. Despite encouraging *ex vivo* autoradiography results, whole-body images were more difficult to interpret due to the proximity with the liver. It is possible a different radionuclide would provide better results; nonetheless this is an interesting application of radiolabelled liposomes as diagnostic tools.

Blood pool imaging by scintigraphy is typically done with ^99m^Tc-radiolabelled autologous erythrocytes or human serum albumin (HSA). A few studies have explored the use of radiolabelled liposomes as a replacement for these products, to simplify the procedure and reduce the risk of contamination from handling blood. Despite results showing good stability of the ^99m^Tc label, sufficient circulation times and biodistributions that were similar to radiolabelled erythrocytes and better than ^99m^Tc-HSA in healthy animals [44,277,278], PEGylated liposomes do not appear to have been further studied for diagnostic purposes in this context. On the other hand, liposome-encapsulated haemoglobin as a substitute for red blood cell transfusions have seen continued interest, and radiolabelling with ^99m^Tc has contributed to establishing their therapeutic potential [[279], [280], [281]]. A PET alternative was developed by Urakami et al. using an ^18^F-based probe that can be incorporated into preformed liposomes [91].

Early studies of liposomes in myocardial infarction used dual-radionuclide radiolabelled liposomes with ^99m^Tc to follow the lipid components and entrapped ^125^I-HSA or ^125^I-polyvinylpyrrolidone in the aqueous compartment [104], or ^3^H-cholesterol and ^99m^Tc-DTPA respectively [282]. This strategy helped assess the integrity of the liposomes in the circulation. Although no imaging was performed at the time, dual-radionuclide SPECT is feasible with the current generation of scanners and should be exploited further. These earlier studies were inconclusive as to the usefulness of liposomes for drug delivery vehicles in myocardial infarction. Liposomes loaded with ^99m^Tc-radiolabelled streptokinase have been used to image thrombi, showing a modest increase in streptokinase accumulation at the target site [283]. The improvement was however too modest to envisage using these liposomes to detect thrombi. Later developments with immunoliposomes [284,285] or other surface modifications to target markers of vascular damage and platelet activation such as lectin-like oxidized low-density lipoprotein receptor-1 (LOX-1), chondroitin sulfate proteoglycans or integrin GP_IIb/IIIa_, have increased the therapeutic potential of liposomes in cardiovascular diseases [286]. For example, Zhang et al. have developed liposomes incorporating ^18^F-fluorodipalmitin in the lipid membrane and coated with an arginine-rich peptide [86]. Dynamic PET imaging demonstrated very rapid accumulation in the heart vasculature and myocardium, which was reduced in mouse models of infarction and reperfusion injury [87]. Such formulations could thus be used both to deliver drugs to the myocardium and diagnose myocardial ischaemia. In this case the rapid kinetics could justify the use of a short-lived radionuclide, but the need to formulate the liposomes after radiosynthesis of the ^18^F-labelled lipid is a significant disadvantage. Remote loading or surface chelation of a radiometal such as ^68^Ga immediately prior to administration would probably have better prospects for clinical translation.

In this light, the approach recently demonstrated by Hood et al. could prove useful for vascular imaging and drug delivery [61]. Liposomes with DTPA-functionalised lipids were radiolabelled with ^111^In prior to administration. Conjugating the liposomes to monoclonal antibodies or single-chain variable fragments (scFvs) targeting the vascular integrins PECAM-1 and ICAM-1 showed higher accumulation in the lungs than non-targeted liposomes. Furthermore, the scFv-conjugated liposomes had increased specific targeting, presumably because the absence of the Fc region reduced the potential for uptake by the RES.

Ishii, Fukuta and colleagues used radiolabelled liposomes to detect cerebral ischemia-reperfusion injury. Liposomes containing a ^125^I-labelled erythropoietin analogue showed increased uptake in the ischemic hemisphere when injected immediately after reperfusion, accompanied by reduction in brain swelling and improved motor scores compared to the non-liposomal drug [108]. However, using a similar liposome radiolabelled with ^18^F but containing no drug, while the increase in ^18^F uptake was higher in the ischemic side of the brain, the signal intensity was similar on both sides, making the use of such liposomes difficult for diagnosis [93]. A potential reason for this was that the increased uptake, presumably due to increased vascular permeability, was offset by the reduction in blood flow. The reasons for the discrepancy between the ^125^I- and ^18^F-labelled formulations are unclear, although we speculate that retention of the EPO analogue and its carrier maybe be different after release. This question could be answered by radiolabelling both the encapsulated drug and the carrier.

### 4.5 Other

Liposomes have been used for topical formulations in ophthalmology, as the layered structure of the mammalian eye presents many barriers to the delivery of therapeutic concentrations of drugs [287]. Cationic liposomes radiolabelled with ^111^In [288] or ^99m^Tc [[289], [290], [291]] had longer pre-corneal residence times after ocular instillation, leading to increased corneal penetration and therapeutic benefits in a rabbit model of cataract. For this application the relatively short half-life of ^99m^Tc was an advantage, as the clearance of the liposomes from the eye occurred within a shorter time frame. From a clinical perspective, however, it would seem safer and probably less expensive to use ocular fluorophotometers [292] and fluorescently labelled liposomes to measure corneal drug delivery, rather than the comparatively burdensome use of radionuclides.

In dermatology, liposomes and other nanoparticulate formulations have been explored as vehicles, usually to increase transdermal drug delivery, with mixed results [293]. On the other hand, one study used ^99m^Tc-labelled liposomes to show an increased skin retention and reduced liver uptake of octyl methoxycinnamate, a photoprotective compound widely used in sunscreens [294].

Liposomes have been investigated as drug delivery vehicles in Alzheimer’s disease, encapsulating an ^18^F-labelled curcumin analogue or using an ^18^F-labelled lipid to track the liposomes and coating the surface with a peptide targeting low-density lipoprotein (LDL) receptors to increase uptake in the brain [295]. By the authors’ own admission, the half-life of ^18^F was too short to match the pharmacokinetics of the liposomes and overall uptake in the brain was low. A superior brain-to-blood ratio was claimed for the LDL-targeting formulations, but this measure can be misleading as it is likely that the very low circulating levels of the liposomes – as a consequence of higher liver and lung uptake values – influence these results. Expressed as SUV, uptake in the brain was in fact no higher than with the non-targeted formulations. Moreover, spatial patterns of ^18^F uptake in the brain did not coincide with the distribution of amyloid plaque. Finally, curcumin and its derivatives have long been known as problematic compounds (PAINS, for pan-assay interference compounds) in drug development, interfering in many assays and one should therefore exercise caution before envisaging to use curcumin analogues for targeting purposes.

Recently, T.M. e Silva et al. reported higher accumulation in the liver of galactosylated liposomes, as a potential drug delivery vehicle for hepatic diseases [53]. Since liposomes generally have high uptake in the liver, it remains to be seen whether this accumulation occurs preferentially in hepatocytes rather than Kupffer cells and whether this approach can have therapeutic benefits. Amin et al. attempted to use nasally delivered liposomes for tetanus vaccination [296]. Scintigraphy imaging showed good deposition and retention of the formulation in the nasal mucosa. This strong interaction with the mucosa may have prevented the liposomes from reaching the lymph nodes, explaining the failure of the formulation to elicit a systemic immune response.

The slower clearance of liposomes compared to small-molecule drugs has been exploited to demonstrate improvements in scanner technology. Wirwarr et al. used ^111^In-labelled liposomes to demonstrate the high sensitivity and temporal resolution of a multiple-pinhole SPECT system [297], and Prior et al. used similar liposomes to improve scatter correction in dual-radionuclide SPECT [298]. Combined with sub-millimetre spatial resolution, such technological advances have played a great part in keeping SPECT radionuclides relevant in preclinical research despite the gradual shift towards the use of PET in the clinic.

In nearly all the studies cited, the sole role of the chelator is to enable the incorporation of a radiometal in a liposome formulation. One study took the opposite approach, using ^99m^Tc to assess the delivery of DTPA for use in chelation therapy [299]. Here, the tropism of liposomes for the liver is beneficial since this is the site of accumulation of toxic heavy metals such as thorium and plutonium.

### 4.6 Personalised medicine

An area of medicine where radiolabelled liposomes – and radiolabelled compounds more generally – hold particular promise, is personalised medicine [8], a concept first proposed by Harrington et al. after observing heterogeneous uptake of liposomes in patients [189]. The ability to predict which patients have a higher probability to respond or not to a given treatment would help tailor the treatments and thus avoid unnecessary treatment to patients who would only experience the adverse effects of drugs. This is particularly relevant for liposomal drugs in oncology. Indeed, the EPR effect, which is the main driver of their accumulation in tumour sites, is known to be highly variable not only between patients, but even between tumours within the same patient [5,300]. Furthermore, many current therapeutic approaches in oncology target the tumour vasculature and can therefore affect tumour uptake of liposomes. Consequently, radiolabelled liposomes could be used to adjust treatments in real time. Previous reviewers of the field commented on potential applications in personalised medicine [301]. It is exciting to see that in recent years, studies have emerged specifically examining the use of radiolabelled liposomes in personalised medicine, which we address below.

Using four different tumour models, Ito et al. observed different responses to liposomal doxorubicin (Doxil®) that correlated with the uptake of drug in the tumours and the degree of tumour vascularisation, both measured by e*x vivo* histology [302]. They then showed that the uptake of ^111^In-labelled liposomes of similar composition correlated with therapeutic efficacy, proving that non-invasive imaging could be used to predict treatment outcome. Similarly, Pérez-Medina et al. used a PEGylated liposomal formulation radiolabelled with ^89^Zr as a companion PET imaging agent for Doxil® [79,80]. There was a strong correlation between the tumour uptake of ^89^Zr measured by PET and the amount of doxorubicin in the tumours, which further translated into a good correlation with tumour growth delay (Fig. 8C) [79]. Despite DFO often being described as a sub-optimal chelator for ^89^Zr, the images at 24 h do not seem to show significant uptake in the bone, proving the stability of the formulation. It is especially noteworthy that this study demonstrated good correlation between the uptake of ^89^Zr and that of other, non-liposomal nanoparticulate formulations such as a nanoemulsion and a PLGA block copolymer nanoparticle, demonstrating that a single formulation could potentially be used as a companion diagnostic for any drug whose uptake is primarily EPR-mediated. This would considerably simplify translation by removing the need to gain regulatory approval for radiolabelled versions of each liposomal or nanoparticulate drug. In a radionuclide therapy model, Hrycushko et *al.* found that taking into account intratumoural heterogeneity for absorbed-dose calculations after local delivery of ^186^Re-labelled liposomes provided better correlation with tumour shrinkage than using the average tumour absorbed dose [303]. While such precise intratumoural distribution analysis is non-trivial, it is expected that future technological improvements will simplify this type of analysis, and will allow to correlate for example the spatial distribution of a radiolabelled drug with the therapeutic outcome with better accuracy than simply using the total amount of drug delivered to the tumour. This is more likely to be achievable with PET imaging because of the increased sensitivity and spatial resolution. It should be kept in mind that it is difficult to replicate the heterogeneity of human tumours with xenograft models, and the use of spontaneous tumour models should be considered when developing personalised medicine approaches [301].

In the clinic, Arrieta et al. administered ^99m^Tc-labelled liposomal doxorubicin in combination with cisplatin to patients with unresectable malignant pleural mesothelioma [304,305]. By dividing patients into groups showing ‘low’ or ‘high’ tumour uptake of ^99m^Tc as measured by SPECT, it was demonstrated that patients with high uptake had significantly higher response rates than those with low uptake. Although the study only included 35 patients, the results suggest that the patients with low uptake could be spared this particular treatment and be switched to alternative treatments. Recently, a body of preclinical and clinical work with ^64^Cu-labelled liposomes has been described by Lee et al. [161,163,164]. Anti-HER2-targeted liposomes loaded with doxorubicin were radiolabelled using 4-DEAP-ATSC as radiolabelling agent (see Section 3.2.4), a method that has the advantage of not requiring prior encapsulation of a copper chelator and is therefore applicable to any liposomal formulation. Imaging data led to classifying patients as having ‘low’ or ‘high’ uptake in the tumour lesion with the lowest uptake, and there appeared to be correlation between progression-free survival and ^64^Cu uptake (Fig. 8D) [163]. Despite the good stability of the ^64^Cu label inside the liposomes *in vitro*, better contrast on the PET images at later time points might have been obtained with the choice of a longer-lived PET radionuclide. Nonetheless, the results warrant further investigations to draw firm conclusions as to the predictive value of liposomal ^64^Cu uptake in tumours. It was then demonstrated in several preclinical models that the uptake of a ^64^Cu-labelled liposomal reporter correlated with the therapeutic efficacy of 3 different liposomal drugs, but not with that of non-liposomal doxorubicin [164], and enabled the monitoring of the effects of an anti-VEGF drug [162]. It was recently shown by Hansen et al. that radiation therapy could affect, positively or negatively depending on the model, liposomal uptake in tumours [141], leading the authors to suggest the use of radiolabelled liposomes to determine which patients would be more likely to benefit from combination treatment with liposomal anticancer drugs.

As mentioned previously in Section 4.2.1, predicting the efficacy of liposomal drugs is probably more accurately done with radiolabelled liposomes than with [^18^F]FDG, because the accumulation of this small-molecule radiotracer in tumours is dependent on metabolic activity rather than on the EPR effect. There is therefore much potential in using radiolabelled liposomes to predict treatment outcome. However, it has long been known that the immune system can recognise PEG chains on liposomes and other nanosized formulations and raise responses that lead to rapid clearance of the carrier upon repeated administration [306]. This phenomenon, known as the accelerated blood clearance (ABC) effect, is mediated in part by anti-PEG immunoglobulin M (IgM) and subsequent activation of the complement system and has been observed in several species [307]. The ABC effect is easily missed in preclinical research because many studies perform single administration of radiolabelled PEGylated liposomes, use immunodeficient models that cannot mount an IgM response, or use liposomes encapsulating drugs – such as doxorubicin or oxaliplatin – that are toxic towards the splenic B cells responsible for anti-PEG IgM production. Recently, the ABC effect was described with tracer doses of ^64^Cu-labelled liposomes in a canine model of spontaneous tumours, which is closer to human cancer than rodent models are, and in immunocompetent rats [140]. This suggested that using small doses (in terms of lipid amounts) of radiolabelled liposomes for EPR determination and patient stratification could backfire by suppressing the effect of subsequent administrations of liposomal drugs. From the pattern of ^64^Cu distribution, it appeared that the accelerated clearance was accompanied by destabilisation of the liposomes. Importantly, the study showed that the ABC effect could be prevented either by using a larger dose of lipids for imaging or by depleting the pool of circulating anti-PEG IgM with a ‘decoy’ administration of liposomes 1 h before administration of the actual tracer or drug, and that the presence of ABC could be detected by measuring anti-PEG IgM levels in blood [140]. Although the role of anti-PEG antibodies is increasingly recognised [308], the importance of the ABC effect in humans is still poorly known. In any case, the results from this study will be important considerations for the design of future clinical trials. To illustrate this, any occurrence of ABC in the ^64^Cu-MM-302 trial [163] would likely not have been observed because the radiolabelled liposomes were administered a few hours after the therapeutic dose, which would presumably have depleted any circulating anti-PEG immunoglobulins. If ABC had occurred to a significant extent, it is likely that the PET images of ^64^Cu-MM-302 uptake would have shown the EPR but not the actual distribution of MM-302. As a consequence, it may be relevant to screen patients for anti-PEG immunoglobulins before using PEGylated drugs or radiotracers. An alternative approach, conceptually similar to the use of humanised antibodies in therapy, may be to use liposomes formed from cell membrane components with reduced immunogenic potential [82].

Another clinical example of the use of a companion diagnostic formulation for liposomal drugs is given in a study using ^99m^Tc-sulfur colloid (TSC) [309]. Palmar-plantar erythrodysesthesia (PPE) is a common side effect in patients treated with liposomal doxorubicin. Under the hypothesis that TSC would be cleared by the RES similarly to liposomal doxorubicin, the aim was to predict the occurrence of PPE by measuring the accumulation of TSC in the patients’ hands. A good correlation was found between TSC levels in the hands and PPE severity, showing TSC could be used to assess individual RES activity and personalize treatments with liposomal doxorubicin accordingly. An equivalent formulation for PET imaging could be similar to the ^64^Cu-labelled albumin described by Rygh et al. [67]. Because these companion diagnostic products do not rely on liposomes for imaging, this approach might also be a useful way to circumvent the accelerated blood clearance problem posed by tracer doses of PEGylated liposomes.

## 5 Conclusions and perspectives

Nuclear imaging is an important research and clinical tool for tracking the biodistribution and pharmacokinetics of liposomal drug delivery nanocarriers. Comparing the two nuclear techniques available, SPECT is perhaps the most versatile for preclinical studies due its high spatial resolution (compared to preclinical PET scanners), the possibility of using several well-established radionuclides and chemistries, and the possibility of imaging specific radionuclide pairs simultaneously. This provides SPECT with the unique ability to track two elements from the liposomal nanomedicine, such as the phospholipids and the drug cargo, *in vivo*. In the clinical setting, however, PET has an advantage from its superior spatial resolution (compared to clinical SPECT scanners), sensitivity and quantification properties. A frequently highlighted disadvantage of nuclear imaging is the need for ionising radiation, which is an important aspect that needs to be considered. It is therefore essential to have access to expert medical physics and health and safety teams capable of monitoring and calculating radiation doses. This will ensure patients and users are safe from unwanted radiation-induced tissue damage. Recent advances in PET technology, and in particular total-body PET, are expected to make a significant impact in this field by allowing whole-body PET imaging of nanomedicines in humans at lower radiation doses (up to 40x lower) and shorter acquisition times [310,311].

Our review highlights the importance of choosing the most appropriate radionuclide and radiolabelling method, as each will have advantages and disadvantages. Ideally, the process of radiolabelling a liposome should result in a product that is stable (both in terms of radiochemical and chemical stability), does not change its overall properties (*e.g.* hydrodynamic size, zeta potential), and importantly informs on its location and concentration *in vivo* within the biological lifetime of the liposome. However, it is important to keep in mind that once *in vivo*, it is not possible to use nuclear imaging to directly assess the integrity of the liposomal drug delivery system, nor the release of the drug cargo. For example, at late imaging time points when liposomes are more likely to have been taken up by macrophages, we should expect that the images represent a mixture of signals from both intact liposomes and the released radionuclide (still attached or not to its corresponding liposome component, depending on the radiolabelling method) as a result of liposome degradation. If the aim is to determine the location of the encapsulated drug rather than the carrier itself, then the preferable option is to encapsulate a radiolabelled drug, if at all possible. It should be kept in mind that nuclear imaging does not allow to directly identify whether the carrier is releasing the drug cargo at the right location. Thus, we can only obtain indirect measures of both *in vivo* stability and drug release kinetics, and in order to do so an understanding and knowledge of the *in vivo* behaviour, biodistribution and pharmacokinetics of the free radionuclide and/or radiolabelled liposome component (*e.g.* phospholipid or encapsulated drug) are essential. In this context, the fact that many metallic radionuclide ions and phospholipids share the same excretion route as liposomes (*i.e.* hepatic), and even show significant tumour uptake (*e.g.* ^64^Cu, ^68^Ga, ^111^In), complicates the assessment of liposomal *in vivo* stability and drug release using PET or SPECT. In contrast, using small metal complexes and radionuclides with well-defined non-hepatic excretion routes such as ^99m^Tc, ^89^Zr and iodide should allow for a clearer distinction between intact liposome and components, and even provide a potential indirect measure for drug release.

Finally, all the research carried out to date in this field highlights that liposomal nanomedicines highly benefit from integration with nuclear imaging techniques. This is particularly important at the clinical stage where human and disease heterogeneity have been shown to be present, and strongly correlated to the target tissue uptake levels and therapeutic efficacy of liposomal nanomedicines. While it may seem at first glance as an additional layer of complexity and an increased upfront cost, incorporating nuclear imaging to inform on the *in vivo* behaviour of liposomal nanomedicines from the early developmental stages to their clinical evaluation stage would help to optimise not only their translational pathway – for example by providing key information when clinical trial endpoints are not achieved – but also allow for imaging-guided personalised nanomedicine treatments. We expect that by gaining a better understanding of the drug distribution and determining early on which patients are more or less likely to benefit from a drug, the number of liposomal nanomedicines achieving significant clinical impact will increase, as well as their clinical efficacy.

## Appendix A Supplementary data

Supplementary material Image 1
