# Supplementary material for: Nuclear imaging of liposomal drug delivery systems: A critical review of radiolabelling methods and applications in nanomedicine
Source: Adv Drug Deliv Rev. 2019 Mar 15;143:134–60. doi: 10.1016/j.addr.2019.05.012 (PMC6866902; doi:10.1016/j.addr.2019.05.012)
Supplement: Supplementary file 1 — Supplementary material [file mmc1.docx]

**Supplementary information for:**

**Nuclear Imaging of Liposomal Drug Delivery Systems: A Critical Review of Radiolabelling Methods and Applications in Nanomedicine**

Francis Man^1§^, Peter J. Gawne^1§^, and Rafael T. M. de Rosales^1^*

^1^ School of Biomedical Engineering & Imaging Sciences

King’s College London, St Thomas’ Hospital, London, SE1 7EH, United Kingdom (UK).

^§^These authors contributed equally.

***Corresponding author**: Rafael T. M. de Rosales, PhD. School of Biomedical Engineering & Imaging Sciences, King’s College London, St Thomas’ Hospital, Westminster Bridge Road, London, SE1 7EH, UK. Telephone: +44(0)20 718 88370. E-mail: [rafael.torres@kcl.ac.uk](mailto:rafael.torres@kcl.ac.uk)

**Methodology:**

In order to identify methods that have been used to radiolabel liposomes to date (2018), we searched PubMed between August and December 2018 using the term ‘liposome’ in combination with the keywords ‘radiolabel(l)ed’, ‘radiolabel(l)ing’, ‘nuclear imaging’, ‘PET, ‘SPECT’, ‘scintigraphy’ and the radionuclides listed in Table 1 (main article). Occasionally, articles not returned by search were found through references within publications. A total of 322 articles (not including reviews, patents, book chapters, conference publications and articles not published in English) were found and analysed to create Figure 1 (main article). Articles were further selected for review based on full-text copies.
